# Aldosterone synthase inhibition: cardiorenal protection in animal disease models and translation of hormonal effects to human subjects

**DOI:** 10.1186/s12967-014-0340-9

**Published:** 2014-12-10

**Authors:** Joël Ménard, Dean F Rigel, Catherine Watson, Arco Y Jeng, Fumin Fu, Michael Beil, Jing Liu, Wei Chen, Chii-Whei Hu, Jennifer Leung-Chu, Daniel LaSala, Guiqing Liang, Sam Rebello, Yiming Zhang, William P Dole

**Affiliations:** Université Paris Descartes, Faculté de Médecine and INSERM/AP-HP Clinical Investigation Center, Georges Pompidou Hospital, Paris, France; Novartis Pharmaceuticals Corporation, East Hanover, NJ USA; Novartis Institutes for BioMedical Research, Cambridge, MA USA; Current address: Golda Och Academy, 1418 Pleasant Valley Way, West Orange, NJ 07052 USA

**Keywords:** Aldosterone, Cortisol, Cushing’s disease, Double-transgenic rat, Eplerenone, Mineralocorticoid, Translational research

## Abstract

**Background:**

Aldosterone synthase inhibition provides the potential to attenuate both the mineralocorticoid receptor-dependent and independent actions of aldosterone. *In vitro* studies with recombinant human enzymes showed LCI699 to be a potent, reversible, competitive inhibitor of aldosterone synthase (*K*_i_ = 1.4 ± 0.2 nmol/L in humans) with relative selectivity over 11β-hydroxylase.

**Methods:**

Hormonal effects of orally administered LCI699 were examined in rat and monkey *in vivo* models of adrenocorticotropic hormone (ACTH) and angiotensin-II-stimulated aldosterone release, and were compared with the mineralocorticoid receptor antagonist eplerenone in a randomized, placebo-controlled study conducted in 99 healthy human subjects. The effects of LCI699 and eplerenone on cardiac and renal sequelae of aldosterone excess were investigated in a double-transgenic rat (dTG rat) model overexpressing human renin and angiotensinogen.

**Results:**

Rat and monkey *in vivo* models of stimulated aldosterone release predicted human dose– and exposure–response relationships, but overestimated the selectivity of LCI699 in humans. In the dTG rat model, LCI699 dose-dependently blocked increases in aldosterone, prevented development of cardiac and renal functional abnormalities independent of blood pressure changes, and prolonged survival. Eplerenone prolonged survival to a similar extent, but was less effective in preventing cardiac and renal damage. In healthy human subjects, LCI699 0.5 mg selectively reduced plasma and 24 h urinary aldosterone by 49 ± 3% and 39 ± 6% respectively (Day 1, mean ± SEM; *P* < 0.001 vs placebo), which was associated with natriuresis and an increase in plasma renin activity. Doses of LCI699 greater than 1 mg inhibited basal and ACTH-stimulated cortisol. Eplerenone 100 mg increased plasma and 24 h urinary aldosterone while stimulating natriuresis and increasing renin activity. In contrast to eplerenone, LCI699 increased the aldosterone precursor 11-deoxycorticosterone and urinary potassium excretion.

**Conclusions:**

These results provide new insights into the cardiac and renal effects of inhibiting aldosterone synthase in experimental models and translation of the hormonal effects to humans. Selective inhibition of aldosterone synthase appears to be a promising approach to treat diseases associated with aldosterone excess.

**Electronic supplementary material:**

The online version of this article (doi:10.1186/s12967-014-0340-9) contains supplementary material, which is available to authorized users.

## Background

The pathogenesis of various forms of adrenal and low-renin hypertension is attributed primarily to the renal effects of excessive aldosterone on sodium retention and potassium elimination [1]. Experimental data have shown that aldosterone can cause cardiac, renal and vascular damage independent of its effects on blood pressure [2]. These experimental data together with growing clinical evidence for the benefits of blocking the actions of aldosterone with mineralocorticoid receptor antagonists (MRAs) in the treatment of heart failure [3-5] provide the rationale for new therapeutic approaches to inhibit aldosterone production. While MRAs are currently the treatment of choice for pathologic conditions due to aldosterone excess [6], they induce a counter-regulatory increase in aldosterone production [7]. This may attenuate the effects of competitive MRAs, and might also contribute to adverse cardiovascular effects through non-genomic mechanisms independent of activation of the mineralocorticoid receptor [8-10]. An alternative approach to inhibiting the effects of aldosterone is blocking the renin-angiotensin system (RAS), which decreases aldosterone production indirectly. However, the resulting fall in plasma aldosterone does not persist in the long term [11,12]. The therapeutic potential of targeting adrenal steroid synthesis to inhibit aldosterone production has not been fully explored due to the lack of selective aldosterone synthase inhibitors (ASIs).

Aldosterone synthase, which is encoded by the CYP11B2 gene, is highly expressed in adrenal gland glomerulosa cells and is expressed at lower levels in other tissues [13,14]. Aldosterone synthase converts 11-deoxycorticosterone (11-DOC) to aldosterone by sequential 11-β hydroxylation, 18-hydroxylation and 18-oxidation [15,16]. Selective inhibition of aldosterone synthase could provide an effective approach to decrease aldosterone production, thus attenuating both receptor-mediated and non-genomic deleterious consequences of aldosterone excess.

We report the *in vitro* and *in vivo* effects of aldosterone synthase inhibition with LCI699 (4-[(5R)-dihydro-5H-pyrrolo[1,2-c]imidazol-5-yl]-3-fluorobenzonitrile phosphate) [17] in rats, non-human primates and humans. We have characterized the enzymatic inhibition and species specificity of LCI699 and have established the relative selectivity of LCI699 for aldosterone synthase over 11β-hydroxylase (encoded by the CYP11B1 gene), which converts 11-deoxycortisol to cortisol and has 93% nucleotide sequence identity with aldosterone synthase [18]. In order to determine the therapeutic potential of an ASI, the effects of LCI699 on cardiorenal damage and survival were assessed in a double-transgenic (dTG) rat model with ectopic overexpression of human renin and angiotensinogen and the results compared with those of the MRA eplerenone. In healthy human subjects, LCI699 selectively inhibited aldosterone synthase at oral doses ≤ 1 mg daily, but lost specificity above the 1 mg dose. Therefore LCI699 is no longer being developed for essential hypertension, and is currently under development at higher, nonselective doses for the treatment of Cushing’s syndrome [19].

## Methods

### *In vitro* enzyme inhibition

#### Experimental design

##### Cell lines and tissue samples

Recombinant human cytochrome P450 (CYP) 11B2 and CYP11B1 enzymes were prepared from the cell lines V79-4 CYP11B2-adrenodoxin-adrenodoxin reductase (AAR) #317 and V79-4 CYP11B1-AAR #618, respectively [20]. Recombinant rat CYP11B2 and CYP11B1 enzymes were prepared similarly. All cell lines were maintained in Dulbecco’s modified Eagle’s medium supplemented with 10% fetal bovine serum, 0.5× antibiotic, 800 μg/mL geneticin and 250 μg/mL hygromycin (double-selection medium; all from Invitrogen, Carlsbad, CA, USA).

Rat adrenal homogenates were prepared from the adrenal glands of male Sprague-Dawley (S-D) rats as described previously [21]. Monkey CYB11B2 and CYB11B1 homogenates were prepared from the adrenal glands of female cynomolgous monkeys. Monkey adrenal gland tissue was minced and homogenized on ice in a glass tissue grinder in 1 mL of ice-cold homogenization buffer per 100 mg tissue (adding 2.7 mmol/L CaCl_2_ and one ethylenediaminetetraacetic acid (EDTA)-free protease inhibitor tablet per 50 mL buffer) [20]. The homogenized material was centrifuged at 450 *g* for 5 min at 4°C, and the supernatant brought to a final glycerol concentration of 5%, flash-frozen in liquid nitrogen, and stored at −80°C until analysis. Aldosterone, cortisol and corticosterone concentrations were quantified using 96-well plate assays (see Additional file 1).

##### CYP11B2 and CYP11B1 enzyme assays

Human CYP11B2 and CYP11B1 assays were performed as described previously [20]. The rat and monkey assays were conducted similarly, using 11-DOC as substrate.

#### Statistical analysis

Concentration–response curves for LCI699 were performed at least three times (and two times for rat homogenate assay). Half-maximal inhibitory concentration (IC_50_) values were derived using a non-linear least-squares curve-fitting program (XLfit; ID Business Solutions Inc., Bridgewater, NJ, USA).

### *In vivo* Ang-II- and ACTH-infusion models

#### Experimental design

##### Animal procedures

All animal procedures were conducted in accordance with an approved Novartis Animal Care and Use Committee protocol and the Guide for the Care and Use of Laboratory Animals as described previously [21]. Male S-D rats (~500 g) were purchased from Taconic Farms (Germantown, NY, USA) and acclimatized in the Novartis vivarium (12 h light/dark cycle; 72°F; 55% relative humidity) for at least 7 days before being used in experiments. Rats were provided normal chow (Harlan Teklad 8604) and water *ad libitum* except for a partial fast before and during an experiment.

Male cynomolgus monkeys (*Macaca fascicularis*) were selected for their cooperation and ability to sit comfortably in chairs and were conditioned to remain in the chairs for extended periods before the studies began. On the day of the experiment, monkeys were removed from their home cages with a pole/collar device (Primate Products, Inc., Woodside, CA, USA), secured in their restraining chairs, and transported to the quiet study room. The monkeys remained in the chair for the duration of the experiment (up to 10 h), during which time an enrichment and feeding regimen was followed.

Both rats and monkeys were surgically instrumented with chronically indwelling femoral venous and arterial catheters to allow intravenous administration of substances and repeated blood sampling. Details of the surgical procedure are outlined in Additional file 1.

##### LCI699 formulation

LCI699 solution was freshly prepared (from powder) before each experiment. In the rat models, LCI699 (free base) was first dissolved in 1.5 molar equivalents of 1 N HCl plus 10 parts of water and then diluted in 3% cornstarch (1 mL/kg volume). In the monkey model*,* LCI699 (phosphate salt) was dissolved in water (1 mL/kg volume). LCI699 was administered by oral (rat and monkey) or nasogastric gavage (monkey). Compound doses in the monkey model are quoted as free base equivalents.

##### Experimental protocol for rat models

Study protocols for the rat models of Ang-II- and ACTH-stimulated aldosterone synthesis followed a published protocol [21]. For the Ang-II-infusion model, an initial loading dose of 300 ng/kg angiotensin II (Ang II) was followed by 100 ng/kg/min intravenous (i.v.) infusion for 9 h. For the ACTH-infusion model, the loading and infusion doses of ACTH were 100 ng/kg and 30 ng/kg/min, respectively. After 1 h of Ang II or ACTH infusion, a blood sample was collected for determining the post-Ang II or ACTH ‘baseline’ (i.e., secretagogue-elevated) plasma aldosterone and corticosterone concentrations. LCI699 was administered at doses of 0.1, 0.3, 1 and 3 mg/kg in the Ang-II-infusion model, and 1, 3, 10, 30 and 100 mg/kg in the ACTH-infusion model. In both models, infusion continued for a further 8 h. Blood samples were withdrawn in heparin (final concentration 15 U/mL) from the arterial cannula at 15 and 30 min, and 1, 2, 3, 4, 5, 6, 7, 8, and 24 h post-dosing. Plasma aldosterone and cortisol were determined by radioimmunoassay and LCI699 by liquid chromatography separation coupled with tandem mass spectrometric detection (LC-MS/MS) (see Additional file 1).

##### Experimental protocol for monkey model

Six monkeys (4.9–8.8 kg) were selected and were divided into two groups of three animals. Experiments were not initiated until after at least 2 weeks of recovery from the catheter/vascular access port (VAP) surgeries. Thirty minutes before the start of the experiment, a Huber needle was inserted transdermally into the VAP for the collection of blood samples and injection of ACTH. Between samplings, catheters/VAPs were flushed with saline and kept patent with 10 U/mL heparin. In all cases, the total blood withdrawn did not exceed 1% of body weight per week, and at least 1 week of recovery was allowed between sequential experiments.

Blood samples (0.3 mL in 15 U/mL heparin) for baseline pharmacokinetic and pharmacodynamic assessments were collected at 0.5 h, 0.25 h and immediately before dosing. LCI699 (5, 15, 50 or 150 μg/kg) or vehicle (water) was administered followed 3 h later by ACTH(1–24) (Cortrosyn®; Amphastar Pharmaceuticals, Inc., Rancho Cucamonga, CA, USA) 3000 ng/kg i.v. in 0.1 mL/kg (over ~2 min). The 3000 ng/kg dose of ACTH was determined from a pilot dose–response experiment, which showed a consistent and maximal stimulation of plasma aldosterone and cortisol. Blood samples were collected at 0.125, 0.25, 0.5, 0.75 and 1 h after ACTH injection to assess the time course of plasma aldosterone and cortisol stimulation. Further blood samples were collected up to 8 h and at 23.5 and 24 h after LCI699/vehicle administration. Between the 8 h and 23.5 h collections, the Huber needles were removed and the monkeys were returned to their home cages. All instrumentation was removed after the last sample at 24 h. Plasma aldosterone and cortisol were determined by radioimmunoassay and LCI699 by LC-MS/MS (see Additional file 1).

#### Statistical analysis

All pharmacokinetic parameters were derived from concentration–time data by non-compartmental analyses and were calculated using WinNonlin (Enterprise, Version 5.2; Pharsight Corporation, Palo Alto, CA, USA).

ACTH responses were defined as the peak plasma aldosterone or cortisol concentration within 1 h after the ACTH injection (i.e., between 3 and 4 h after LCI699 administration), expressed as a percentage of the corresponding monkey’s response in the vehicle control experiment. Half-maximal effective dose (ED_50_) values for the ACTH-stimulated responses were estimated by linear regression of these individual percentage peak responses versus logarithmic LCI699 dose. Half-maximal effective concentration (EC_50_) values were calculated in a similar fashion by separate linear regression of the percentage peak responses with the logarithmic LCI699 plasma concentrations at 3 h and 4 h, and the mean 3-h and 4-h values.

Student’s paired *t*-test was used to evaluate pairwise comparisons of responses after LCI699 or vehicle in a given monkey. Data and statistical analyses were conducted with Microsoft Excel (Version 2007, Microsoft, Redmond, WA, USA). All differences were considered statistically significant at a two-sided *P* value ≤ 0.05.

### Double-transgenic rat models

#### Experimental design

To determine the effects of inhibiting aldosterone synthase in an experimental model of rapidly progressive RAS-driven hypertension with cardiac and renal damage, we used dTG rats overexpressing human renin and angiotensinogen, as previously described [22]. dTG rats are characterized by high circulating levels of human renin, Ang II and aldosterone, left ventricular (LV) hypertrophy, and rapidly progressive cardiac and renal disease with moribundity or death occurring on average by 2 months of age.

In order to study the effects of aldosterone inhibition on survival in older dTG rats with established cardiorenal disease, we modified the model by treating male dTG rats during their developmental stage (3–8 weeks of age) with the angiotensin-converting enzyme (ACE) inhibitor enalapril. We determined that pre-treatment of male dTG rats with enalapril at a dose of 10 mg/kg/day (40 mg/L in drinking water) from 3 to 8 weeks of age prolonged median survival by approximately 4 months. These rats developed progressive cardiac and renal impairment, allowing evaluation of the effects of aldosterone synthase inhibition with LCI699 in dTG rats with pre-established cardiorenal disease and high mortality. A similar model was recently reported in dTG rats by St-Jacques *et al.* [23].

dTG rats were bred from their respective single transgenic lines at Novartis under a license agreement with the Max-Delbruck-Centrum fur Molekulare Medizin (Berlin-Buch, Germany). Age-matched male S-D rats were bred by Taconic Farms (Germantown, NY, USA). This background strain for the dTG rats served as healthy controls. Rats were maintained on a 12-h light/dark cycle at 72°F with 55% relative humidity and were provided normal food (Harlan Teklad 8604) and water *ad libitum*.

##### Experimental rat protocols

Rats were treated with LCI699 (phosphate salt) dissolved in drinking water or eplerenone in chow. LCI699 concentrations (ranging from 12 to 400 mg/L) were adjusted for changes in water consumption to achieve the targeted doses of 3–100 mg/kg/day. At the nominal doses of 3, 10, 30 and 100 mg/kg LCI699, the actual measured doses were 4 ± 0, 10 ± 1, 28 ± 3 and 65 ± 4 mg/kg, respectively. Administration of LCI699 in drinking water dose-dependently and proportionally increased total plasma concentration of LCI699 from 58 nmol/L at the lowest dose to 2000 nmol/L at the highest dose.

The maximum effective dose of eplerenone for prolongation of survival was 30 mg/kg, which was the dose chosen to assess the effects of mineralocorticoid receptor blockade on cardiorenal structure and function. Two rats were excluded from all data analysis owing to severe hematuria and moribundity on the scheduled take-down day.

In studies in young dTG rats, water consumption was measured and urine collected for renal biomarkers 24 hours before the terminal experiment. On the final experimental day, rats were anesthetized to allow for echocardiographic examination. A carotid artery was then cannulated for direct recording of arterial pressure and heart rate. Blood (aortic puncture) and organ/tissue samples were collected, and the animal was euthanized.

In experiments with older dTG rats (treated with enalapril for 5 weeks), urine collections and echocardiographic examinations were conducted as above but systolic arterial pressure was estimated in conscious rats by tail plethysmography (Model# 56-1, IITC Life Science Inc., Woodland Hills, CA, USA) using appropriate tail cuff sensors (B63), and restrainer (size 80), at a constant chamber temperature of 30°C.

Echocardiographic images were acquired with a Vivid 7 (GE Healthcare, Milwaukee, WI, USA) instrument and an M12L (GE Healthcare) linear probe (14 MHz) with appropriate frame rates (M-mode: 164.8 frames per second (fps); 2D mode: 210.3 fps; TDI mode: 365.5 fps). Images were analyzed offline with EchoPAC™ Dimension software (GE Healthcare).

In the survival studies, dTG rats were euthanized if they became moribund. Moribundity was defined as a decrease in body weight of ≥ 20% from “baseline” or two consecutive days of observable hematuria. The study endpoint was the age of the animal at death or at moribundity requiring euthanization.

##### Sample processing for morphometric evaluation

Hearts were perfused *in situ* with KCl (50 mmol/L), which quickly arrested the heart and depolarized the myocytes allowing for a more standardized and reproducible assessment of myocardial cell size. Hearts were fixed in 10% formalin. Myocyte cross-sectional area was assessed on sections cut at the mid-papillary level and stained with 1:100 FITC wheat-germ agglutinin (Vector Lab, catalog # FL-1021). Digital images were taken from the LV free wall, interventricular septum and papillary muscle regions. Image analysis of round cells (~500 per heart) on cross-section was performed to estimate mean cell size.

##### Sample processing for clinical chemistry

Serum and urinary electrolytes (Na^+^, K^+^, and Cl^−^) and creatinine and blood urea nitrogen (BUN) were assessed with a Hitachi 917 chemistry analyzer. Urinary albumin concentration was measured with a commercially available enzyme immunoassay (EIA) kit (catalog # A05102; SPI-BIO, Montigny Le Bretonneux, France) according to the manufacturer’s instructions. Plasma aldosterone and corticosterone concentrations and LCI699 concentrations were assessed as previously described (also see Additional file 1).

### Human pharmacokinetic–pharmacodynamic and safety studies

#### Experimental design

##### Study population

Healthy male volunteers, 18–45 years of age and with a body mass index of 18–28 kg/m^2^ were eligible for this study. Subjects had to be non-smokers with normal hepatic and renal function for inclusion. Subjects included in the multiple-dose phase of the study had to have a normal cortisol stimulation test (cortisol > 580 nmol/L or > 21 μg/dL) on Day –2. All subjects provided informed written consent before participating in any study procedures. The study was conducted at two centers in the Netherlands and one in Germany and was approved by the Independent Ethics Committee and/or Institutional Review Board for each study center (Stichting Beoordeling Ethiek Biomedisch Onderzoek, Assen, Netherlands; Ethics Committee of the Land, Berlin, Germany) and was conducted in accordance with Good Clinical Practice and the ethical principles of the Declaration of Helsinki.

##### Study design

This was a randomized, double-blind, placebo-controlled, interwoven single- and multiple-ascending-dose study. In the single-dose phase, five cohorts of eight subjects each were enrolled. Each cohort received one of five LCI699 doses (3, 10, 30, 100 or 200 mg) in ascending order. Subjects were randomized to LCI699 or placebo in a 6:2 ratio. Assessments were made throughout the treatment period, and at an end of study assessment 7 days post-dose.

The multiple-dose phase involved four cohorts, each of 18 subjects. After inhibition of the cortisol stimulation test was observed with the first few subjects in the 10 mg multiple-dose cohort, the protocol was amended to study lower doses of 0.5, 1 and 3 mg for the multiple-dose phase. In each cohort, subjects were randomized to once-daily doses of LCI699, placebo or the MRA eplerenone 100 mg in a 12:3:3 ratio for 14 days. Subjects were placed on a controlled sodium restricted diet from the day before dosing until 72 h post-dose and were housed at the study center for this period. Until Day 7, the diet was ~50–60 mEq Na^+^/~50–60 mEq K^+^. On the morning of Day 8, potassium in the diet was increased to ~100 mEq. Based on urinary electrolyte values obtained from placebo subjects on Day 7 and Day 14 respectively, the actual diet was ~95 ± 63 mEq Na^+^/46 ± 12 mEq K^+^ for the first week and ~86 ± 42 mEq Na^+^/69 ± 25 mEq K^+^ for the second week.

##### LCI699 formulation

LCI699 (phosphate salt) was provided as hard gelatin immediate-release capsules (0.5, 5 and 50 mg) for oral administration.

##### Pharmacokinetic assessments

Blood samples for pharmacokinetic assessment were obtained pre-dose and at regular intervals up to 72 h post-dose in the single-dose phase of the study. In the multiple-dose phase, blood samples were taken on Day 1 and Day 14 at regular intervals up to 36 and 72 h post-dose, respectively, and pre-dose on Days 3, 4, 7 and 11. Blood samples (2 or 4 mL) were collected into an EDTA containing tube, plasma extracted and samples frozen at –70°C until analysis. LCI699 concentrations in plasma were analyzed by LC-MS/MS (see Additional file 1).

##### Pharmacodynamic assessments

Pharmacodynamic assessments included the measurement of aldosterone, plasma renin activity (PRA), cortisol and 11-deoxycortisol during both phases of the study. In addition, ACTH and 11-DOC were measured during the multiple-dose phase. During the single-dose phase, blood samples for pharmacodynamic assessments were collected pre-dose and at 1, 2, 4, 8, 12 and 24 h post-dose. During the multiple-dose phase, samples were collected pre-dose and at 1, 2, 4, 8 and 12 h post-dose on Days −1 (baseline), 1, 7, 8 and 14, as well as pre-dose on Days 2 and 9, and at 24, 48 and 72 h after the final dose (i.e., on Days 15, 16 and 17). In addition, subjects underwent a cortisol stimulation test on Days –2, 6 and 13; An ACTH analog was injected 2 h post-dose and blood samples were collected prior to, and at 30 and 60 min after, the ACTH injection. Aldosterone and PRA levels were measured by radioimmunoassay and cortisol and 11-deoxycortisol levels by LC-MS/MS (see Additional file 1).

##### Safety and tolerability assessments

All adverse events were recorded during the study period. In addition, vital signs and body weight were measured daily, while blood hematologic and chemistry profiles, urinalysis, physical condition and 12-lead electrocardiograms were monitored regularly throughout the study.

#### Statistical analysis

All subjects with quantifiable pharmacokinetic measurements were included in the pharmacokinetic data analysis. Pharmacokinetic parameters were analyzed separately for the single- and multiple-dose phases and were determined using non-compartmental analyses. A power model (area under curve [AUC] or maximum plasma concentration [C_max_] = α * Dose^β^) was used to evaluate dose proportionality of LCI699 exposure after single- and multiple-dose administration [24].

All subjects with evaluable pharmacodynamic measurements were included in the analysis, and data from the two study phases were analyzed separately. An analysis of covariance (ANCOVA) with treatment as factor and baseline value as covariate was performed to evaluate changes from baseline in pharmacodynamic parameters at each time point. Least-squares mean differences and associated 95% CIs were calculated for comparison of LCI699 and eplerenone groups with placebo.

An analysis of variance (ANOVA) with treatment as the classification factor was performed on orthostatic changes (standing-supine) in pre-dose systolic blood pressure, diastolic blood pressure, and heart rate measurements for each of Days 1, 7, and 14 separately. Each LCI699 dose and eplerenone treatment were compared with the placebo treatment (subjects pooled across all cohorts) within the ANOVA framework.

An ANCOVA with treatment as the classification factor and baseline as the covariate was performed on change from baseline (Day 1 pre-dose) to Day 7 and Day 14 for pre-dose supine and standing systolic blood pressure (SBP), diastolic blood pressure (DBP), and heart rate and for orthostatic changes (standing–supine). Each LCI699 dose and the eplerenone treatment were compared with the placebo treatment within the ANCOVA framework.

## Results

### *In vitro* enzyme inhibition

**Figure 1:**
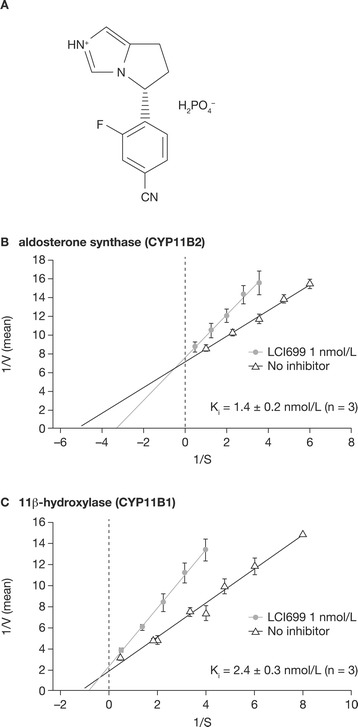
**Structure of LCI699** (**A**) **and** ***in vitro*** **inhibition of (B) aldosterone synthase (CYP11B2) and (C) 11β-hydroxylase (CYP11B1). (A)** shows LCI699 phosphate salt. **(B)** shows Lineweaver–Burk plots of inhibition by LCI699 of human recombinant aldosterone synthase (CYP11B2) and 11β-hydroxylase (CYP11B1). Intersection of lines indicates competitive inhibition of both enzymes by LCI699 1 nmol/L. Values are mean ± SEM (n = 3 for all determinations). CYP, cytochrome P450.

LCI699 dose-dependently inhibited the activity of recombinant human aldosterone synthase (IC_50_ = 0.7 nmol/L) with 3.6-fold selectivity over 11β-hydroxylase (IC_50_ = 2.5 nmol/L) (Table 1). Lineweaver–Burk plots (Figure 1) showed that LCI699 is a reversible competitive inhibitor of recombinant human aldosterone synthase (*K*_i_ = 1.4 ± 0.2 nmol/L, mean ± SEM) and, at higher concentrations, of 11β-hydroxylase (*K*_i_ = 2.4 ± 0.3 nmol/L).

**Table 1:** ***In vitro*** **selectivity of LCI699 in human, rat and monkey enzymes**

**Assay**	**Aldosterone synthase (CYP11B2) IC**_**50**_ **, nmol/L**	**11β-hydroxylase (CYP11B1) IC**_**50**_ **, nmol/L**	**Selectivity ratio** ^**a**^ **(CYP11B2:CYP11B1)**
Recombinant human enzyme	0.7 ± 0.01 (6)	2.5 ± 0.1 (4)	3.6
Recombinant rat enzyme	160 ± 9 (3)	410 ± 5 (3)	2.6
Rat adrenal homogenate	802 (2)	3045 (2)	3.8
Monkey adrenal homogenate	12 ± 1 (6)	62 ± 5 (6)	5.2

Table shows selectivity ratios for LCI699 for inhibition of aldosterone synthase (CYP11B2) over 11β-hydroxylase (CYP11B1). All values are mean ± SEM (n) except for rat adrenal homogenate where only mean values from two experiments are presented.

^a^Selectivity ratio is defined as the ratio of IC_50_ values obtained in the CYP11B2 and CYP11B1 assays.

CYP, cytochrome P450; IC_50_, half maximal inhibitory concentration.

*In vitro* enzymatic studies using rat recombinant enzymes showed that LCI699 was approximately 230-fold less potent at inhibiting rat aldosterone synthase than the human enzyme (Table 1). Nevertheless, LCI699 had similar weak selectivity for recombinant rat aldosterone synthase and 11β-hydroxylase (2.6-fold difference) compared with that for the recombinant human enzymes.

In monkey adrenal homogenates, the IC_50_ of LCI699 for aldosterone synthase was 17-fold higher than that for the human recombinant enzyme, but was 67-fold lower than the IC_50_ measured in rat adrenal homogenates. Selectivity for aldosterone synthase over 11β-hydroxylase was 5.2-fold in monkey adrenal homogenates.

In summary, the relative species rank order of LCI699 potency for inhibiting aldosterone synthase was human > monkey > rat, whereas the 3- to 5-fold selectivity for aldosterone synthase over 11β-hydroxylase was similar across these species.

### Animal pharmacokinetic and pharmacodynamic studies

#### In vivo effects of LCI699 in rat and monkey models of adrenal hormone stimulation

Pharmacokinetic data for LCI699 after single oral administrations in rats (0.1–3 mg/kg in the Ang II model, and 1–100 mg/kg in the ACTH model; Additional file 2A–B) showed that LCI699 was rapidly absorbed (time to maximum plasma concentration [*t*_max_] 0.3–2.4 h) with a terminal elimination half-life (*t*_½_) of 2–5 h. Over the tested dose range, the pharmacokinetics of LCI699 were dose-proportional. Plasma protein binding was low (35.9%).

The effects of LCI699 on aldosterone and corticosterone synthesis were assessed in two *in vivo* rat models. Adrenal hormones were stimulated with either exogenous Ang II or ACTH. Oral administration of LCI699 dose-dependently inhibited the increase in plasma aldosterone concentrations stimulated by Ang II or ACTH, with an apparent plateau effect above 1 mg/kg for Ang II stimulation and 10 mg/kg for ACTH stimulation. Maximal reductions in plasma aldosterone from baseline of 80% were reached approximately 2 h after dosing (Additional file 3A–B).

**Figure 2:**
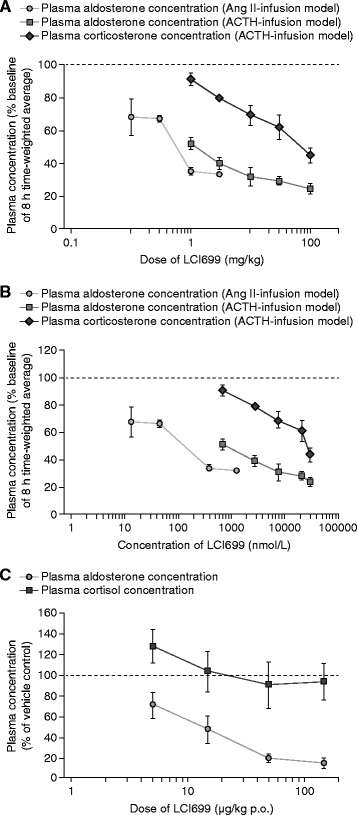
**Effect of LCI699 on Ang-II- and ACTH-stimulated hormone release in rat and monkey** ***in vivo*** **models. (A)** Dose–response plot and **(B)** exposure–response plot (8-h time-weighted average concentrations as percentage of baseline values) of effect of LCI699 on Ang-II stimulated plasma aldosterone and adrenocorticotropic hormone (ACTH)-stimulated plasma aldosterone and corticosterone in rats and **(C)** dose–response plot of effect of LCI699 on ACTH-stimulated plasma aldosterone and cortisol concentrations in monkeys. Values are mean ± SEM. Number of evaluable animals was n = 3 per dose group.

Dose–response relationships for LCI699 in the two rat models are shown in Figure 2A. The ED_50_ for inhibiting Ang-II-stimulated aldosterone response was 0.6 mg/kg. The corresponding half-maximal effective plasma LCI699 concentration (EC_50_ = 127 nmol/L; Figure 2B) was similar to the *in vitro* IC_50_ (160 nmol/L) for inhibiting rat recombinant aldosterone synthase. In the ACTH-infusion model, the LCI699 ED_50_ and EC_50_ values for inhibiting stimulated aldosterone (1.1 mg/kg; 771 nmol/L) and corticosterone (73 mg/kg; 36 μmol/L) responses resulted in dose and exposure selectivity ratios of 65 and 47, respectively (results not shown).

#### Monkey ACTH-stimulation model

Pharmacokinetic data for LCI699 after single oral drug administrations in monkeys (15–150 μg/kg) showed that LCI699 was rapidly absorbed (*t*_max_ 0.9–3.5 h), with a *t*_½_ of 1–2 h (Additional file 2C) and dose-proportional pharmacokinetics. Plasma protein binding was low (26.6%).

Oral administration of LCI699 (5–150 μg/kg) 3 h prior to ACTH injection dose-dependently inhibited the ACTH-stimulated increase in plasma aldosterone concentration (Additional file 3C). The highest LCI699 dose (150 μg/kg) caused approximately a 90% decrease in response compared with the vehicle control. Plasma aldosterone levels returned to baseline 24 h after dosing. No significant inhibition of ACTH-stimulated cortisol synthesis was observed with any dose of LCI699 tested (5–150 μg/kg), indicating *in vivo* selectivity of LCI699 for inhibition of aldosterone over cortisol synthesis in the monkey (data not shown).

The estimated LCI699 ED_50_ for inhibition of ACTH-stimulated plasma aldosterone was 13 μg/kg (Figure 2C). Plasma concentrations of LCI699 below the assay limit of quantification prevented calculation of an accurate EC_50_ value; however, it could be estimated at less than 1 nmol/L, considerably lower than the *in vitro* IC_50_ for inhibition of aldosterone synthase in monkey adrenal homogenates.

### Animal disease model studies

#### Development of hyperaldosteronism and cardiorenal disease in young dTG rats

**Figure 3:**
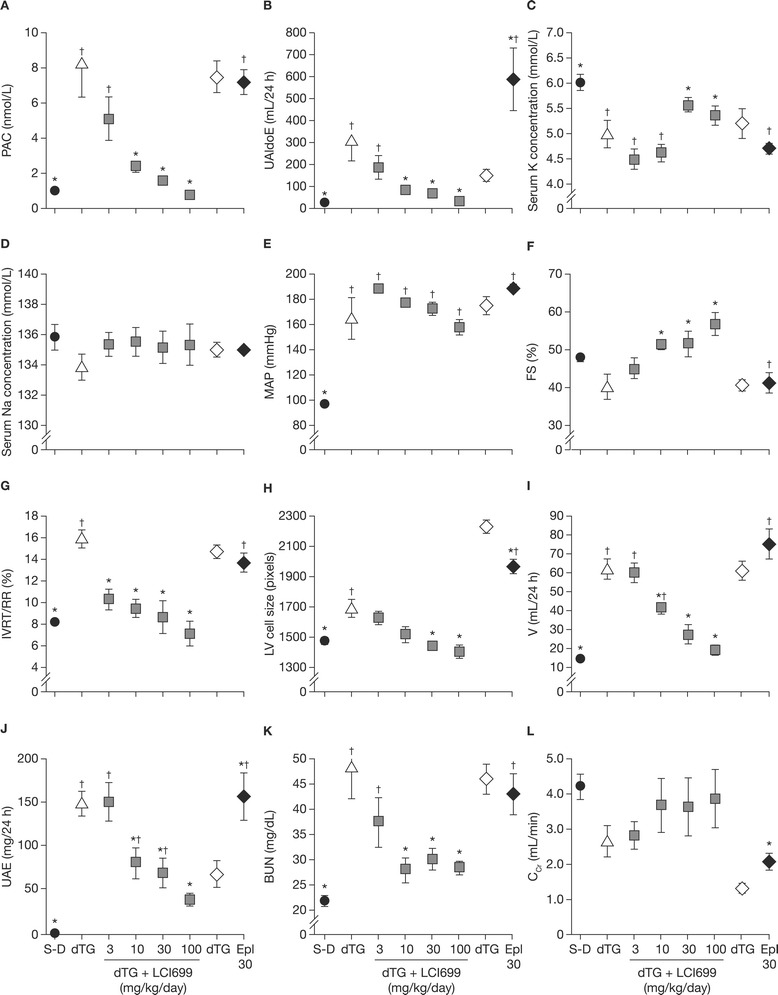
**Effect of LCI699 and eplerenone on adrenal hormones, serum electrolytes, cardiac and renal parameters in dTG rats. (A)** Plasma aldosterone concentrations (PAC); **(B)** urinary aldosterone excretion (UAldoE); **(C)** serum potassium; **(D)** serum sodium; **(E)** mean arterial pressure (MAP); **(F)** LV fractional shortening (FS); **(G)** LV isovolumic relaxation time (IVRT) as a percentage of R-R interval; **(H)** LV myocardial cell size; **(I)** 24-h urine volume (V); **(J)** 24-h urinary albumin excretion (UAE); **(K)** blood urea nitrogen (BUN) and **(L)** creatinine clearance (C_Cr_). Values are shown for double-transgenic (dTG) rats (triangle), dTG rats treated with LCI699 (3, 10, 30 or 100 mg/kg/day in drinking water) or eplerenone (Epl, 30 mg/kg/day in chow) from 3 to 7 weeks of age (square) and in control Sprague-Dawley (S-D) rats (circle). Eplerenone vehicle controls are depicted separately from LCI699 vehicle controls. Measurements were taken at 7 weeks of age. Values are mean ± SEM. Number of evaluable animals was n = 6 per dose group, with the exception of S-D rat controls (n = 11). *P < 0.05 vs dTG rats. ^†^ *P* < 0.05 vs S-D rats.

Compared with age- and strain-matched control S-D rats, dTG rats had elevated plasma aldosterone concentrations (8-fold) and 24 h urinary aldosterone excretion (15-fold) (Figure 3A–B). Plasma corticosterone was not significantly different between dTG rats and control S-D rats (data not shown). Serum potassium was lower in dTG rats compared with S-D rats (Figure 3C–D) consistent with hyperaldosteronism. Beginning at 5 weeks of age, dTG rats developed progressive hypertension, LV hypertrophy, impaired cardiac function (Figure 3E–H), and ventricular arrhythmias, culminating in death between 7 and 9 weeks of age. dTG rats were polydipsic and polyuric with a 4-fold higher urine volume flow compared with S-D rats (Figure 3I). Impaired renal function also developed in dTG rats, as evidenced by elevated serum BUN levels and increased urinary albumin excretion (Figure 3J–K).

#### Effects of LCI699 and eplerenone on adrenal hormone, cardiac and renal abnormalities in dTG rats

**Figure 4:**
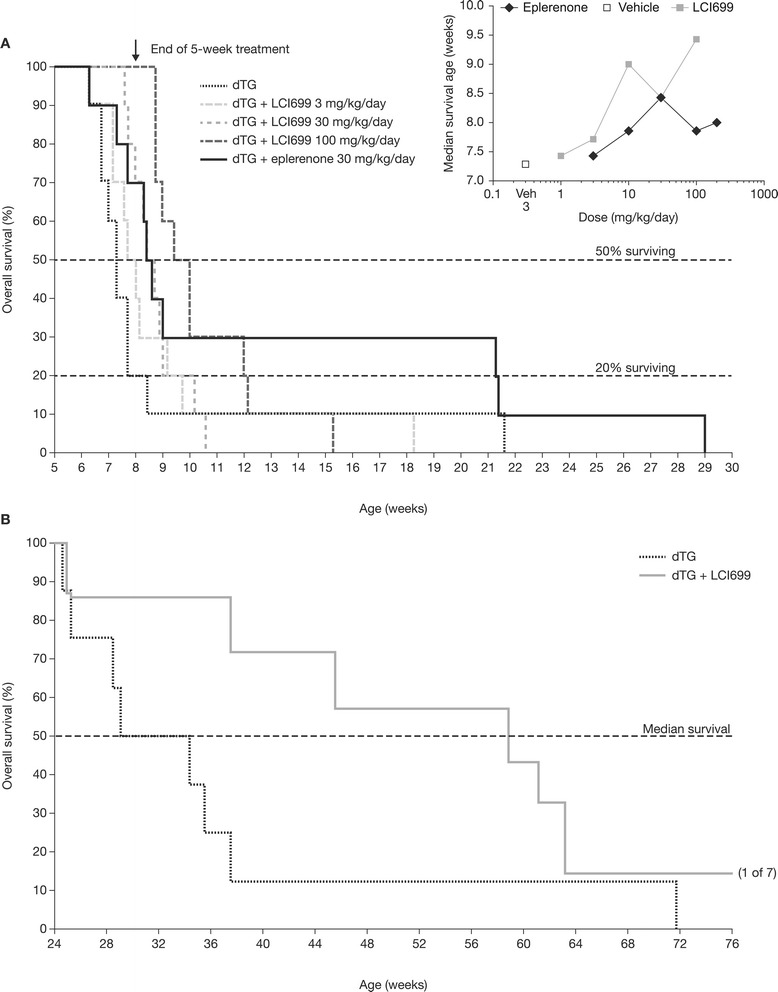
**Effect of LCI699 on survival in young dTG rats and older dTG rats with established cardiorenal disease. (A)** Kaplan–Meier survival curves for young, male double-transgenic (dTG) rats (control) and dTG rats treated with LCI699 (3, 30 or 100 mg/kg/day) or eplerenone (30 mg/kg/day) in drinking water from 3 to 8 weeks of age. An additional group was treated continuously with 30 mg/kg/day for the duration of the study. Inset shows dose–median survival curves for LCI699 and eplerenone. **(B)** Kaplan–Meier survival curves for older male dTG rats with established cardiorenal disease who were pre-treated with enalapril (10 mg/kg/day in drinking water) from 3 to 8 weeks of age to slow the progression of disease. At 24 weeks of age, dTG rats had developed cardiorenal disease and were randomized to a control group (n = 8) or an LCI699 treatment group (30 mg/kg/day in drinking water) until moribundity or death. LCI699 prolonged survival in older dTG rats with established cardiorenal disease.

Treatment with LCI699 dose-dependently normalized plasma aldosterone concentration (EC_50_ = 50 nmol/L; *P* < 0.05) and also reduced urinary aldosterone and corticosterone excretion (Figure 3A–B). In contrast, eplerenone (30 mg/kg/day, a dose shown to maximally prolong survival [Figure 4, inset]) had no effect on plasma aldosterone of dTG rats and significantly increased (*P* < 0.05) urinary aldosterone excretion (data not shown), confirming its biologic activity as an MRA. LCI699 corrected serum potassium in a dose-dependent manner (Figure 3C) whereas eplerenone had no effect.

Significant differences in cardiovascular effects were observed between LCI699 and eplerenone. Although LCI699 tended to decrease mean arterial pressure slightly, neither LCI699 nor eplerenone normalized arterial pressure (Figure 3E). LCI699 dose-dependently increased fractional LV shortening, normalized LV isovolumic relaxation time to RR (IVRT/RR) ratio and myocardial cell size (*P* < 0.05; Figure 3F–H) and reduced LV weight (not shown). In contrast, eplerenone (30 mg/kg) had no significant effect on either fractional shortening or IVRT/RR ratio, reduced but did not normalize myocardial cell size (Figure 3E–H) and had no effect on LV weight (data not shown).

Significant differences in renal effects were also observed between LCI699 and eplerenone. Treatment of dTG rats with LCI699 dose-dependently normalized BUN levels and urinary albumin excretion, water intake (data not shown) and urine output (*P* < 0.05). In contrast, eplerenone had no significant effect on BUN or urine output, while urinary albumin excretion was slightly increased, *P* < 0.05 vs vehicle) (Figure 3I–L).

Measures of LV cell size and weight, kidney weight, LV function (systolic and diastolic) and renal function, were highly correlated with plasma aldosterone concentrations for pooled data from all five groups of dTG rats (controls and LCI699-treated; Additional file 4).

#### Effect of LCI699 on survival in dTG rats with or without established cardiorenal disease

Aldosterone synthase inhibition significantly prolonged survival in dTG rats without established cardiorenal disease. In the control group, 9 of 10 dTG rats died by 8.4 weeks of age. LCI699 prolonged survival in a dose-dependent manner (*P* < 0.01) starting at 10 mg/kg/day (Figure 4A). Eplerenone also significantly prolonged survival (*P* < 0.01) at doses > 10 mg/kg/day. There was no statistically significant difference between the dose-survival curves for eplerenone (3 –300 mg/kg/day) and LCI699 (1–100 mg/kg/day) (*P* = 0.2). However, eplerenone appeared to affect early deaths less than LCI699, and the maximum median survival time with eplerenone (8.4 weeks) was less than that observed with LCI699 (9.5 weeks; Figure 4 inset). Although the precise cause of death in the dTG rats could not be ascertained, 40% required euthanasia owing to moribundity and 60% died unexpectedly and suddenly. There were no significant differences in mode of death between control dTG rats and those treated with either LCI699 or eplerenone.

LCI699 (30 mg/kg/day in drinking water; n = 7) also prolonged survival in dTG rats with established cardiorenal disease. The disease model employed dTG male rats 24 weeks of age pre-treated with the ACE inhibitor enalapril from 3 to 8 weeks of age to reduce early post-natal mortality. Figure 4B shows Kaplan–Meier curves for the control dTG group and the dTG group treated with LCI699. In all, 4 of 8 control dTG rats died by 29 weeks of age and 7 of 8 died by 38 weeks of age. LCI699 treatment tended to prolong median survival by 23 weeks (*P* = 0.07). Whereas all of the control dTG rats died unexpectedly and suddenly, only two of the seven LCI699-treated rats died suddenly.

### Human pharmacokinetic and pharmacodynamic studies

#### Subjects

Ninety-nine healthy male volunteers were randomized and received LCI699 or placebo; 37 in the single-dose phase and 62 in the multiple-dose phase. Demographic characteristics were similar across treatment groups. Mean ages were 21–31 years in the single-dose groups and 32–36 years in the multiple-dose groups, and almost all subjects (96%) were Caucasian. Baseline characteristics of subjects who entered the multiple-dose phase are shown in Table 2.

**Table 2:** **Baseline characteristics of healthy subjects (human study, multiple-dose phase)**

**Parameter**	**LCI699 0.5 mg**	**LCI699 1 mg**	**LCI699 3 mg**	**Eplerenone**	**Placebo**
	**n = 12**	**n = 12**	**n = 12**	**n = 10**	**n = 11**
Age, years	33.0 ± 6.0	32.1 ± 7.2	34.2 ± 8.1	32.5 ± 5.2	35.8 ± 7.8
Body weight, kg	80.5 ± 6.1	76.1 ± 6.2	76.8 ± 6.9	79.2 ± 6.0	82.8 ± 8.6
Supine heart rate, bpm	57 ± 5	63 ± 10	61 ± 5	62 ± 9	64 ± 8
Supine blood pressure, mmHg	128/75 ± 8/7	124/73 ± 11/8	123/75 ± 8/4	124/73 ± 9/6	124/74 ± 9/6
Plasma aldosterone, pg/mL	204.8 ± 78.7	186.4 ± 74.5	236.1 ± 85.6	244.9 ± 80.3	224.2 ± 83.5
Plasma cortisol, ng/mL	120.5 ± 35.4	113.5 ± 28.2	112.9 ± 34.7	121.6 ± 46.1	103.4 ± 46.7
Plasma ACTH, pg/mL	21.9 ± 6.9	26.0 ± 9.6	30.7 ± 16.6	26.8 ± 14.1	22.1 ± 14.6
Plasma renin activity, ng/mL/h	1.24 ± 0.59	1.03 ± 0.65	1.00 ± 0.32	1.83 ± 1.36	1.30 ± 1.04
Plasma sodium, mmol/L	140.8 ± 1.4	140.9 ± 1.2	140.8 ± 1.4	141.2 ± 1.3	140.9 ± 2.1
Plasma potassium, mmol/L	4.5 ± 0.3	4.3 ± 0.2	4.5 ± 0.1	4.3 ± 0.3	4.3 ± 0.3
Serum creatinine, μmol/L	94.5 ± 6.5	93.1 ± 10.0	91.5 ± 6.3	90.6 ± 6.9	92.1 ± 4.4
Blood glucose, mmol/L	4.9 ± 0.5	4.9 ± 0.3	5.2 ± 0.5	5.2 ± 0.5	4.9 ± 0.7

Values are mean ± SD unless otherwise stated.

Thirteen subjects were discontinued from the study during the multiple-dose phase. All five subjects receiving LCI699 10 mg in the multiple-dose cohort were discontinued from the study (two after 10 doses due to an abnormal ACTH stimulation test as pre-specified in the protocol, and three after three doses for safety precautions based on results of the ACTH test). No additional subjects were enrolled in the 10 mg cohort and because of this protocol change three subjects in the 10 mg cohort who were receiving eplerenone (n = 1) or placebo (n = 2) were also discontinued. Five subjects in the 3 mg cohort (three receiving LCI699 3 mg, one receiving eplerenone and one receiving placebo) were discontinued after nine or 10 doses (as per protocol) due to changes in body weight and vital signs (> 2 kg decrease in body weight and > 20 bpm increase in heart rate [HR] upon standing). The three subjects receiving LCI699 3 mg also had plasma sodium levels < 135 mEq/L at the time of discontinuation, which returned to > 135 mEq/L within 1 to 2 days. No subjects were discontinued in the LCI699 1 mg cohort.

#### Safety and tolerability

LCI699 was well tolerated. Adverse events reported with single LCI699 doses up to 200 mg were all mild in intensity except for one event of postural dizziness, which was of moderate intensity. Headache and dizziness (without hypotension) were the most common events during the single-dose phase, and there was no dose-dependence of either the frequency or severity of reported adverse events.

During the multiple-dose phase of the study, only one adverse event (abnormal ACTH-stimulation test with LCI699 10 mg) was considered severe owing to the extent and duration of cortisol suppression. However, the subject showed no signs or symptoms of adrenal insufficiency or hypotension. The frequency of minor adverse events with LCI699 (headache, dizziness and fatigue) was similar to that for eplerenone and placebo, and showed no dose-dependence.

Multiple-dose treatment with LCI699 3 mg was associated with pre-specified markers of hypoaldosteronism in three subjects who were discontinued as per protocol; these effects included mild hyponatremia (plasma sodium concentration < 135 mmol/L), postural tachycardia and decreased body weight (> 2 kg). There were no instances of hypotension with LCI699 at the 3 mg dose. Two other subjects in this cohort (one receiving eplerenone and one receiving placebo) also exhibited postural tachycardia and weight loss, and were discontinued as per protocol.

#### Pharmacokinetics of LCI699

**Figure 5:**
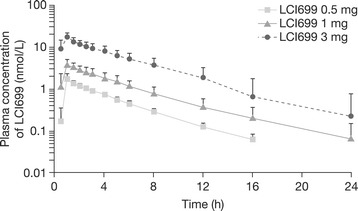
**Pharmacokinetic profile of LCI699 following once-daily oral administration in humans.** Plasma concentration–time profile for LCI699 (mg dose of phosphate salt) on Day 14 following once-daily oral administration in healthy human volunteers for 14 days. Values are mean ± SD. Number of evaluable subjects for LCI699 0.5, 1 and 3 mg were n = 12, 12 and 9 respectively.

Mean plasma concentration–time profiles for LCI699 0.5–3 mg on Day 14 following once-daily oral administration for 14 days are shown in Figure 5. LCI699 was rapidly absorbed with C_max_ reached ~1 h after dosing (mean *t*_max_, 1.00–1.25 h). The *t*_½_ of LCI699 was about 4 h after both single and multiple doses (mean *t*_½_ 3.4–4.8 h across doses). Consistent with the relatively short half-life of LCI699, trough plasma concentrations reached steady state on Day 2, and the ratio of AUC values on Day 14 and Day 1 showed no significant accumulation of LCI699 (accumulation index 0.85–1.32). Plasma protein binding of LCI699 was low (26.6%), similar to that observed in rats and monkeys.

Plasma exposure to LCI699 was approximately dose-proportional over the dose range of 0.5–3 mg. Based on multiple-dose data, the exponent β was 1.13 (90% CI, 1.00–1.25) for AUC_0–24_ and 1.16 (90% CI, 1.07–1.26) for C_max_, indicating minimal deviation from true dose-proportionality.

Evaluation of higher LCI699 doses up to 200 mg in the single-dose phase of the study showed similar values to the lower doses for *t*_½_ (~4 h) and *t*_max_ (~1 h). Over the dose range 3–200 mg, LCI699 AUC_0–24_ exhibited an exponent β of 1.29 (90% CI, 1.24–1.34; Additional file 5).

#### Pharmacodynamic effects of aldosterone synthase inhibition: single-dose phase

##### Plasma and urinary aldosterone

Single doses of LCI699 (3–200 mg) reduced plasma aldosterone concentration 2–24 h post-dose compared with placebo, with a maximal reduction of 60–78% from baseline at 12 h. LCI699 at these doses also reduced urinary aldosterone concentration by 68–81% from baseline. There was no apparent pattern of dose-dependence for either parameter within this dose range, indicating that the maximum effect was achieved at the 3 mg dose (results not shown).

##### Plasma and urinary cortisol

LCI699 at doses of 3–100 mg did not significantly alter plasma cortisol; a single dose of LCI699 200 mg caused a 19% decrease in plasma cortisol 24 h post-dose (*P* = 0.029 vs placebo). Significant reductions in 24-h urinary cortisol (34–42%) were observed following single LCI699 doses of 30, 100 or 200 mg (vs 33% increase with placebo relative to time-matched baseline; *P* ≤ 0.001), but with no clear dose-dependence (results not shown).

##### Cortisol precursor

Levels of the cortisol precursor 11-deoxycortisol increased with LCI699 at doses ≥ 100 mg (942% and 584% with 100 mg and 200 mg, respectively; *P* < 0.001 vs placebo), likely due to off-target inhibition of 11-β hydroxylase (results not shown).

Given the lack of dose-dependence in effects of LCI699 on aldosterone and observed effect on cortisol synthesis with single doses of LCI699 ≥ 30 mg, the multiple-dose phase was designed to evaluate doses of 0.5–10 mg.

#### Pharmacodynamic effects of aldosterone synthase inhibition: multiple-dose phase

##### Plasma and urinary aldosterone

**Figure 6:**
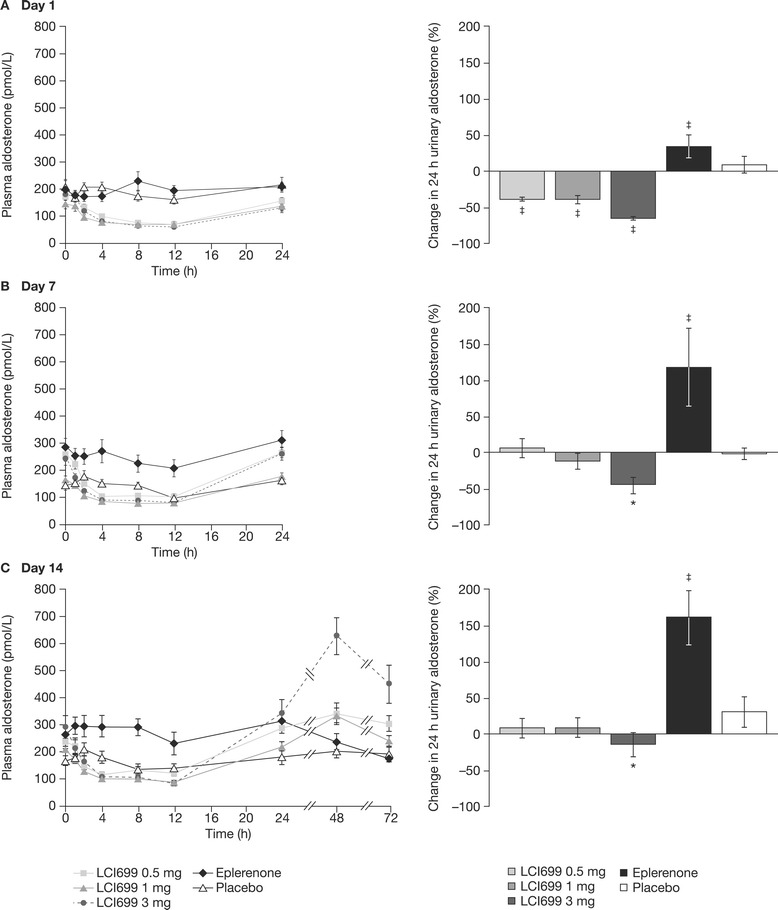
**Effect of LCI699 on plasma and urinary aldosterone concentrations (human study, multiple-dose phase).** Mean plasma aldosterone concentrations and percentage change in 24-hour urinary aldosterone from time-matched baseline on **(A)** Day 1, **(B)** Day 7 and **(C)** Day 14 of once-daily treatment with LCI699 (0.5, 1 or 3 mg), eplerenone (100 mg) or placebo in healthy human volunteers. Values are mean ± SEM. Number of evaluable subjects for LCI699 0.5, 1, 3 mg, eplerenone and placebo were (plasma) n = 12, 12, 12, 10 and 11 respectively, and (urine) n = 12, 9, 11, 9, 11. *P < 0.05, ^‡^ *P* < 0.001 vs placebo.

Compared with the small increase (37%) in plasma aldosterone concentration observed with placebo from time-matched baseline (Day −1), LCI699 reduced plasma aldosterone concentration at 12 h on Day 1 (0.5 mg, –49%; 1 mg, –47%; 3 mg, –63%; all *P* < 0.001 vs placebo); plasma aldosterone concentrations returned to baseline after 24 h (Figure 6A). No further reduction in plasma aldosterone was observed following the 10 mg dose of LCI699 (data not shown). On Days 7 and 14, LCI699 (0.5–3 mg) also reduced plasma aldosterone from pre-dose baseline levels (Figure 6B–C). Mean pre-dose plasma aldosterone levels increased, however, from Day 1 to Day 14 during multiple-dose administration of LCI699 (1 mg and 3 mg) from 400–502 pmol/L (144–181 pg/mL) on Day 1 to 458–719 pmol/L (165–259 pg/mL) on Day 7 and 580–813 pmol/L (209–293 pg/mL) on Day 14. This was due to an increase in aldosterone occurring at some time during the 12–24 h post-dose nocturnal period (not investigated). Following the last dose of LCI699 3 mg on Day 14, plasma aldosterone levels showed a rebound (peak increase at 48 h post-dose) that continued during the post-treatment washout phase (72 h after the final 3 mg dose; Figure 6C).

On Day 1, all three doses of LCI699 reduced 24 h urinary aldosterone levels from baseline (0.5 mg, –39%; 1 mg, –39%; 3 mg, –66%; all *P* < 0.001 vs placebo; Figure 6A). On Day 7, the changes in 24 h urinary aldosterone levels with LCI699 were diminished (1 mg, −12%; 3 mg, −44%) relative to Day 1 and were significantly different from placebo only for the 3 mg dose (Figure 6B). On Day 14, reductions in 24 h urinary aldosterone following LCI699 administration showed little difference compared with placebo except at the 3 mg dose (Figure 6C).

On Day 1, eplerenone 100 mg had no effect on plasma aldosterone concentration, but increased 24 hour urinary aldosterone by 34% (*P* < 0.001 vs placebo). On Days 7 and 14, eplerenone 100 mg significantly increased both plasma aldosterone (46% on Day 7 and 63% on Day 14) and 24 h urinary aldosterone levels (118% and 161%, on Days 7 and 14, respectively) (*P* < 0.001 vs placebo for all analyses).

##### Plasma and urinary cortisol

LCI699 at doses of 0.5–3 mg had no significant effect on plasma or 24 h urinary cortisol on Day 1, 7 or 14 (data not shown; see Additional file 6 for Day 1). On Day 7, one subject treated with LCI699 10 mg showed a large (> 70%) reduction in 24 h urinary cortisol, while the second subject showed no change.

##### Aldosterone and cortisol precursors

Baseline trough plasma concentrations of the aldosterone precursor, 11-DOC, were near the lower limit of quantification (151 pmol/L [50 pg/mL]) across the groups on Day 1. While there was no change in trough 11-DOC levels on Day 7 after aldosterone synthase inhibition with LCI699 0.5 or 1 mg, LCI699 3 mg led to a 228% increase in 11-DOC levels from baseline (*P* < 0.001 vs placebo; data not shown).

Without ACTH stimulation, an increase in cortisol precursor (11-deoxycortisol) was observed at LCI699 doses ≥ 3 mg, although only the 10 mg dose was statistically significant. LCI699 10 mg was associated with increases in Day 6 pre-dose plasma concentrations of the cortisol precursor, 11-deoxycortisol of 508% from baseline vs −16.5% with placebo (*P* < 0.001). Consistent with this, no compensatory increases in trough or peak ACTH levels were observed at doses ≤ 3 mg although LCI699 10 mg increased pre-dose ACTH levels compared with baseline by 193% (*P* < 0.001 vs placebo). Pre-dose plasma ACTH measurements on Day 6 showed small decreases from baseline levels with placebo (−12.3%) and with LCI699 0.5–3 mg (0.5 mg, −14.5%; 1 mg, −31.4%; 3 mg, −15.6%), which did not differ significantly from placebo.

##### ACTH stimulation test: aldosterone and cortisol

**Figure 7:**
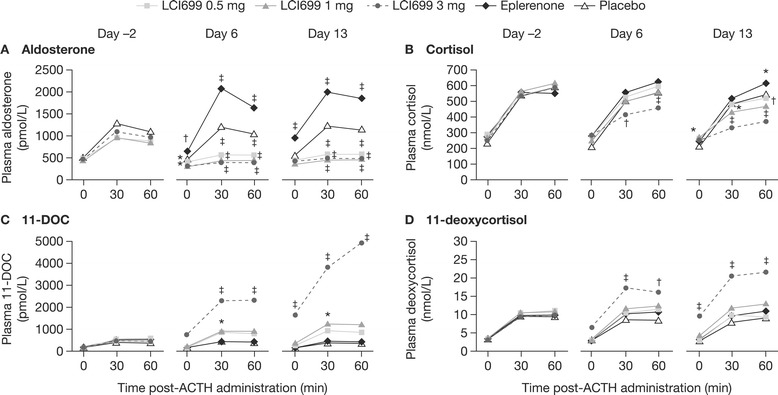
**Dose- and time-dependent effects of LCI699 on ACTH-stimulated aldosterone, cortisol and precursors (human study, multiple-dose phase).** Mean plasma concentrations of **(A)** aldosterone, **(B)** cortisol, **(C)** the aldosterone precursor 11-deoxycorticosterone (11-DOC), and **(D)** the cortisol precursor 11-deoxycortisol, at 0, 30 and 60 min after adrenocorticotropic hormone (ACTH) administration at baseline (Day –2) and on Days 6 and 13 of once-daily treatment with LCI699 (0.5, 1 or 3 mg), eplerenone (100 mg) or placebo in healthy human volunteers. Values are means. Number of evaluable subjects for LCI699 0.5, 1, 3 mg, eplerenone and placebo were (Day -2 and 6) n = 12, 12, 12, 10 and 9 respectively, and (Day 13) n = 12, 12, 9, 8 and 8 respectively. *P < 0.05, ^†^ *P* < 0.01, ^‡^ *P* < 0.001 vs placebo.

The ACTH stimulation test was performed 2 h post-LCI699 dose at the observed *t*_max_. For ACTH-stimulated aldosterone levels (Figure 7A), LCI699 0.5–3 mg resulted in peak inhibition of 41–64% from baseline (Day −2) on Day 6 (vs 7% reduction with placebo; *P* < 0.001). By contrast, treatment with eplerenone 100 mg caused a progressive increase in pre-ACTH aldosterone levels 2 h post-dose (increase vs Day −2 pre-ACTH levels; Day 6, 29%; *P* ≤ 0.01 vs placebo) (Figure 7A). In eplerenone-treated subjects, ACTH stimulation on Day 6 resulted in a 63% increase in peak aldosterone levels.

LCI699 3 mg reduced ACTH-stimulated cortisol levels on Day 6 (peak 22% reduction from baseline; *P* < 0.05 vs placebo) (Figure 7B), whereas lower doses had no significant effect (LCI699 0.5 mg, peak 1.8% reduction from baseline; 1 mg, 11%). LCI699 10 mg further suppressed ACTH-stimulated cortisol (*P* < 0.05 vs placebo) on Days 6 and 10, which reversed completely 4–9 days after discontinuing LCI699.

##### ACTH stimulation test: precursors of aldosterone and cortisol synthesis

LCI699 1 mg and 3 mg increased ACTH-stimulated levels of the aldosterone precursor 11-DOC on Day 6 (maximal increases from baseline 95% and 407%, respectively) (Figure 7C). LCI699 0.5 mg and eplerenone had no significant effect on ACTH-stimulated 11-DOC levels. Consistent with the time-dependent effects of LCI699 on aldosterone synthesis, increases in ACTH-stimulated 11-DOC levels with LCI699 1 mg and 3 mg were larger on Day 13 than Day 6 (809% and 168%, respectively; Figure 7C).

LCI699 3 mg also increased ACTH-stimulated levels of the cortisol precursor 11-deoxycortisol on Day 6 (peak 82% increase from baseline; *P* < 0.001 vs placebo), with a larger effect on Day 13 (peak 114% increase; *P* < 0.001; Figure 7D). Eplerenone and lower doses of LCI699 had no significant effect on cortisol precursors on Day 6 or Day 13.

#### Plasma and urinary sodium and potassium

Aldosterone synthase inhibition with LCI699 induced a rapid natriuresis on Day 1 of treatment (Table 3). The natriuretic effect of LCI699 0.5 mg was similar to that observed with eplerenone 100 mg and significantly greater than that with placebo. LCI699 tended to decrease plasma sodium slightly (2–4 mmol/L) after 6 days’ administration of the 3 mg dose. In six LCI699-treated subjects, plasma sodium levels were at or below 135 mmol/L (two and four subjects receiving LCI699 0.5 mg and 3 mg, respectively); there were no such changes observed in subjects receiving eplerenone or placebo. No further changes in sodium excretion were observed with LCI699 and eplerenone compared with placebo on Day 7 or 14, indicating that a steady-state sodium balance was achieved by Day 7.

**Table 3:** **Effect of LCI699 on plasma and urinary sodium and potassium (human study, multiple-dose phase)**

	**LCI699 0.5 mg**		**LCI699 1 mg**		**LCI699 3 mg**		**Eplerenone**		**Placebo**	
	**n**	**Mean**	**n**	**Mean**	**n**	**Mean**	**n**	**Mean**	**n**	**Mean**
*Plasma sodium, mEq/L*										
Baseline	12	139.3	12	140.1	12	140.6	10	139.9	11	139.6
Δ Day2^a^	12	+0.23	12	−0.59	12	−0.73*	10	−0.64	11	+0.26
Δ Day7^b^	12	−1.71^‡^	12	−0.11	12	−2.87^‡^	10	+0.31	11	+0.41
Δ Day14^c^	12	+0.41	12	−0.01	12	−0.45	10	−0.07	11	+0.35
*Plasma potassium, mEq/L*										
Baseline	12	4.19	12	4.03	12	4.13	10	3.97	11	4.14
Δ Day2^a^	12	+0.20	12	+0.08	12	+0.40^‡^	10	+0.08	11	0.00
Δ Day7^b^	12	+0.25^‡^	12	+0.12*	12	+0.28^‡^	10	−0.02	11	−0.05
Δ Day14^c^	12	+0.05	12	−0.06	12	+0.22*	10	−0.09	11	−0.06
*Urinary sodium, mEq/24 h*										
Baseline	12	52.3	9	109.3	11	60.7	9	66.5	11	70.1
Δ Day 1^a^	12	+45.2^†^	9	+76.3^‡^	11	+64.0^‡^	9	+53.8‡	11	+5.6
Δ Day 7^b^	12	+3.6	9	+44.0	11	+22.2	9	+16.2	11	+16.7
Δ Day 14^c^	12	−25.5	12	−3.6	12	−17.2	10	−24.0	9	−12.2
*Urinary potassium, mEq/24 h*										
Baseline	12	38.7	9	42.5	11	49.1	9	46.4	11	44.8
Δ Day 1^a^	12	+9.2	9	+9.7	11	+2.9	9	+5.2	11	+8.6
Δ Day 7^b^	12	+12.8	9	+17.4*	11	+12.2	9	+6.4	11	+1.6
Δ Day 14^c^	12	+20.2	12	+4.2	12	+37.6*	10	+3.7	9	+17.5

^a^Δ values are comparisons of Day 1/Day 2–baseline (Day −1); ^b^Δ values are comparisons of Day 7– baseline (Day −1); ^c^Δ values are comparisons of Day 14– Day 7. Mean changes (last observation carried forward) were derived from an analysis of covariance model with baseline value as a covariate. Based on urinary electrolyte values obtained from placebo subjects on Day 7 and Day 14 respectively, the actual diet was ~95 ± 63 mEq Na^+^/46 ± 12 mEq K^+^ for the first week and ~86 ± 42 mEq Na^+^/69 ± 25 mEq K^+^ for the second week.

*P < 0.05, ^†^ *P* < 0.01, ^‡^ *P* < 0.001 vs placebo.

There was a slight increase in plasma potassium with LCI699 3 mg, on Day 1 (+0.40 mEq/L), Day 7 (+0.28 mEq/L) and Day 14 (+0.22 mEq/L) of treatment and a small increase in urinary potassium excretion on Day 14 (+37.6 mEq/24 h); changes that were not observed in the other groups.

#### Plasma renin activity

**Figure 8:**
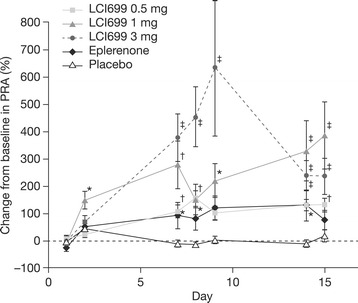
**Effects of LCI699 on plasma renin activity (PRA) (human study, multiple-dose phase).** Percentage change in PRA from time-matched baseline during once-daily treatment with LCI699 (0.5, 1 or 3 mg), eplerenone (100 mg) or placebo for 14 days in healthy human volunteers, and at 24, 48 and 72 h after the final dose. Values are mean ± SEM. Number of evaluable subjects for LCI699 0.5, 1, 3 mg, eplerenone and placebo were n = 12, 12, 12, 10 and 9 respectively. *P < 0.05, ^†^ *P* < 0.01, ^‡^ *P* < 0.001 vs placebo.

Aldosterone synthase inhibition with LCI699 led to increases in PRA, reflecting RAS counter-regulation (Figure 8). Baseline PRA ranged from 0.65 to 1.83 ng/mL/h across treatment groups, indicating mild stimulation of RAS activity likely due to sodium restriction. All doses of LCI699 significantly increased PRA compared with placebo on Day 7 (Figure 8). The increase in PRA from baseline with LCI699 0.5 mg (106%) was similar to that observed with eplerenone 100 mg (92%), while larger increases were observed with LCI699 1 mg (277%) and 3 mg (377%). Significant increases in PRA compared with baseline were also observed with all doses of LCI699 on Day 14 (130%, 327% and 238% increases with LCI699 0.5, 1 and 3 mg, respectively) and persisted for 72 h after the last 3 mg dose. Compared with the effect of LCI699, the increase in PRA with eplerenone on Day 14 was 130%, and returned toward baseline 48 h post-dose.

#### Blood pressure, heart rate and other laboratory parameters

Compared with placebo, no consistent changes in supine systolic or diastolic blood pressure or in heart rate were observed following single or multiple doses of LCI699 or eplerenone (not shown). There were no significant changes in ECG, urinalysis or in hematologic, hepatic or other laboratory parameters.

## Discussion

This is the first report of the effects of pharmacologic inhibition of aldosterone synthase in healthy human subjects. Results obtained with the ASI LCI699 indicate that the hormonal and renal effects of blocking the aldosterone pathway in healthy animals translate to humans. In healthy volunteers, once-daily oral dosing with LCI699 0.5 mg selectively reduced plasma and urinary aldosterone, which was associated with natriuresis and an increase in PRA. LCI699 prolonged survival in a rat disease model induced by ectopic overexpression of human renin and angiotensinogen, and was more effective than the MRA eplerenone in preventing cardiac and renal damage. These results support the therapeutic potential of inhibiting aldosterone synthase in diseases characterized by excessive aldosterone production.

Characterization of LCI699 was performed using *in vitro* assays and *in vivo* models in the rat and monkey. LCI699 showed distinct differences between species; it was at least 200-fold less potent in inhibiting rat recombinant adrenal enzymes (and even less potent using rat adrenal tissue homogenates) and ~20-fold less potent in inhibiting monkey adrenal homogenate enzymes compared with human recombinant enzymes. The *in vivo* Ang-II- and ACTH-stimulation models used to characterize the pharmacodynamic effects of LCI699 in rats and monkeys predicted the human oral dose potency and the short duration of action of LCI699 for inhibition of aldosterone synthase. Thus, LCI699 dose-dependently inhibited ACTH-stimulated increases in aldosterone with an EC_50_ in the low nanomolar range in monkeys, consistent with the low *in vitro* IC_50_ (0.7 nmol/L) obtained with recombinant human aldosterone synthase. Both the rat and monkey *in vitro* data showed relative selectivity of LCI699 for aldosterone synthase over 11β-hydroxylase (3–5-fold) similar to that observed with the human enzymes. However, both the rat and monkey *in vivo* models overestimated the selectivity of LCI699 in humans. In the rat, *in vivo* ED_50_ values showed 47-fold selectivity of LCI699 for aldosterone synthase over 11β-hydroxylase, while in the monkey there was no attenuation of the ACTH-stimulated cortisol response by LCI699 even at doses above the ED_50_, which impaired cortisol synthesis in human subjects. Cai and co-workers evaluated the effects of LCI699 (administered intravenously at doses in the range 0.003–3 mg/kg) on ACTH-stimulated aldosterone and cortisol in rhesus monkeys on a low-salt diet [25]. They showed no effect of LCI699 on ACTH-stimulated cortisol at doses of 0.003–1 mg/kg, similar to our findings in cynomologus monkeys with LCI699 (administered orally at doses of 0.005–0.15 mg/kg). In rhesus monkeys, only the highest, 3 mg/kg i.v. dose of LCI699 reduced ACTH-stimulated cortisol. Thus, the results of Cai and co-workers [25] are consistent with our finding of a relatively high *in vivo* selectivity of LCI699 for inhibition of synthesis of aldosterone over cortisol in monkeys, which was not observed in humans. Overall, these results demonstrate the utility and limitations of using different species for *in vitro* and *in vivo* characterization of ASIs and emphasize the importance of early human investigations for selection of ASI drug candidates based on potency and selectivity [26].

In the dTG rat model, inhibition of aldosterone synthase with LCI699, which dose-dependently blocked increases in aldosterone, prevented development of cardiac and renal functional abnormalities (indicated by changes in LV fractional shortening, IVRT/RR ratio, LV weight, BUN, urinary albumin excretion), and reversed hormonal and electrolyte abnormalities associated with elevated levels of aldosterone. It is noteworthy that the organ protective effects of aldosterone synthase inhibition with LCI699 occurred in the absence of any major reduction in blood pressure. The correlations between plasma aldosterone concentration and observed cardiac and renal structural and functional abnormalities in the present study (Additional file 4) provide additional evidence in support of direct deleterious effects of aldosterone on the heart and kidneys. In addition, dTG rats were polydipsic and polyuric (4-fold higher urine volume flow compared with S-D rats). While Ang-II-induced hypertension with resulting pressure natriuresis has been suggested as the cause for the increase in urine volume [27], our observation that LCI699 normalized urine volume in dTG rats with little effect on blood pressure indicates that other mechanisms may play a more important role such as impaired renal concentrating ability associated with hyperaldosteronism and hypokalemia [27]. The finding that aldosterone synthase inhibition exerts organ protective effects independent of blood pressure lowering also suggests that blood pressure should not be used as the sole surrogate marker of cardiorenal risk reduction in early clinical testing of ASIs.

LCI699 prolonged survival in both young dTG rats and older animals with established hypertension and cardiorenal disease. The MRA eplerenone at a dose of 30 mg/kg body weight also improved survival in a young dTG rat model, but increased plasma and urinary aldosterone and had little effect on several key markers of cardiorenal damage that were normalized by LCI699. Our results confirm and extend those of Fiebeler *et al.*, who showed that the aldosterone synthase (CYP11B2) inhibitor FAD286 or adrenalectomy attenuated cardiorenal damage and reduced mortality in young dTG rats [28]. Collectively, these data suggest that aldosterone, in addition to Ang II and hypertension, is an important mediator of cardiac and renal damage in the dTG rat model. Synergistic genomic and non-genomic interactions between aldosterone and Ang II have been described in both *in vitro* and *in vivo* studies, and have been implicated in vascular inflammation, fibrosis and remodeling [29,30]. Although corticosterone, the physiologic glucocorticoid in rodents, and cortisol in humans have the same affinity for the mineralocorticoid receptor as aldosterone and are present in higher concentrations than aldosterone [31], plasma corticosterone levels in dTG rats were not higher than those observed in age-matched S-D rats and so it is unlikely that corticosterone contributed significantly to the cardiorenal disease observed in the dTG rat model. It is notable that enalapril pre-treatment extended median survival by approximately 4 months in the dTG rat. This may be due to the cardiorenal protective effects of reducing Ang II levels by inhibiting ACE that may influence survival beyond what can be achieved by inhibition of aldosterone synthesis. Regardless of possible synergy between Ang II and aldosterone, our results indicate that inhibiting aldosterone synthase alone is sufficient to prevent development and slow progression of cardiac and renal disease in the dTG rat model.

LCI699 was synthesized as the first orally active aldosterone synthase inhibitor for human use based on the chemical structure of FAD286A, the dextroenantiomer of the aromatase inhibitor fadrozole. Studies in recombinant hamster fibroblasts expressing human CYP11B1 and CYP11B2 showed that LCI699 was a more potent inhibitor of aldosterone synthesis than FAD286A (IC_50_ 0.2 vs 0.8 nmol/L) and more selective for inhibition of aldosterone vs cortisol synthesis (selectivity factor 15-fold vs 7.9-fold) [32]. However, selectivity of LCI699 for aldosterone synthase over 11β-hydroxylase was comparable to that for FAD286 based on recombinant human CYP11B1 and CYP11B2 enzymatic assays [20]. LCI699 and FAD286A also exhibited broadly similar selectivity for inhibition of aldosterone synthase over 11β-hydroxylase in the *in vivo* rat ACTH stimulation model (selectivity factor by exposure 47-fold for LCI699 in the present study, vs 48-fold for FAD286A in a previous study) [21]. Preclinical studies in dogs showed that LCI699 exhibited greater oral bioavailability than FAD286A (96% vs 33% for a 1 mg/kg oral dose), a longer half-life (0.6–3.6 h vs 0.9–1.2 h), and more potent inhibition of Ang II-stimulated aldosterone release (EC_50_ 41 nmol/L vs 136 nmol/L) (D. Rigel, personal communication). FAD286A was tested in a similar dTG rat model to that used in the present study [28]. The effects of FAD286A (4 mg/kg in the diet from Weeks 4–7) on albuminuria and cardiac hypertrophy appeared less pronounced than those that we observed with LCI699; however, subsequent studies using higher doses of FAD286A found comparable pharmacologic benefits of LCI699 and FAD286A (D. Rigel, personal communication).

It is important to consider the potential differences in clinical benefit between inhibiting the synthesis of aldosterone with an ASI and inhibiting the mineralocorticoid receptor with an MRA. These have been reviewed in detail, with specific reference to LCI699, by Azizi and colleagues [33]. One issue of particular clinical relevance is hyperkalemia, which is a well-known adverse effect of MRAs and other inhibitors of RAS activity. It is, however, uncertain if the different mechanism of action of ASIs compared with MRAs will alter the risk of hyperkalemia. Some insights may be gained by comparing the phenotypes of mineralocorticoid receptor- and aldosterone synthase-deficient mice generated by gene targeting. Mineralocorticoid receptor-deficient mice show a partial defect of the amiloride-sensitive Na^+^ channel (ENaC) in the colon and kidney [34,35]. The phenotype is characterized by strongly enhanced fractional excretion of sodium resulting in hyponatremia and severe hyperkalemia with reduced fractional excretion of potassium compared with wild type mice, reflecting a transport defect in the principal cells of the collecting duct. In contrast, mice with genetic deficiency of aldosterone synthase demonstrate modest hyperkalemia with normal urinary potassium excretion [36]. Interestingly, an increase in potassium excretion on Day 7 was observed in our study of healthy subjects following administration of LCI699, but not eplerenone. One possible explanation for this difference is that 11-DOC (increased by LCI699 but not eplerenone) which been shown to stimulate potassium excretion in the renal collecting tubules, may have a relative potassium-sparing effect without promoting sodium retention [37,38]. The clinical relevance of this remains uncertain, however, as a study conducted in patients with mild-to-moderate hypertension showed a significant dose-response relationship for LCI699 (total daily dose range 0.25 mg to 1 mg) and serum potassium levels; moreover, changes in serum potassium concentration with LCI699 were similar in magnitude to those observed with the MRA eplerenone (50 mg twice daily) [39].

The clinical benefits of MRAs have been demonstrated previously [3-5]. However, the efficacy of MRAs may be limited by the fact that the renal loss of sodium and retention of potassium induced by MRAs stimulate a dose-dependent increase in renin, Ang II and aldosterone [7]. This may counteract the beneficial actions of MRA treatment both by competition of increased aldosterone at the level of the mineralocorticoid receptor and by stimulating non-genomic (mineralocorticoid receptor-independent) effects of aldosterone [40]. On the other hand, cardiomyocyte mineralocorticoid receptors can be occupied by physiologic glucocorticoids, and under conditions of tissue damage cortisol can become a mineralocorticoid receptor agonist [41], mimicking the experimental effects of aldosterone through a mechanism that would not be affected by an ASI. Our results that cardiomyocyte cell size was normalized by LCI699 in the dTG rat suggest that such a mechanism plays little, if any, role in this experimental model.

The majority of untreated dTG rats in our studies experienced unexpected and sudden death, whereas the majority of LCI699-treated animals eventually became moribund, necessitating euthanasia. Although the mode of death is uncertain, it is possible that inhibiting aldosterone synthase may reduce mortality in part by preventing sustained ventricular arrhythmias and subsequent hemodynamic deterioration [42]. The clinical relevance of such findings is suggested by the Randomized ALdactone Evaluation Study (RALES), which demonstrated that in patients with congestive heart failure, the addition of spironolactone to standard medical therapy significantly reduced both cardiac mortality and sudden cardiac death [3].

This study provides the first comprehensive characterization of the hormonal effects of inhibiting aldosterone synthase in humans. In the clinical study, moderate sodium restriction was used to activate the renin-angiotensin pathway and stimulate baseline aldosterone levels, and thereby facilitate detection of dose-dependent decreases in aldosterone and counter-regulatory increases in renin. Sodium and potassium intake were strictly controlled while subjects were housed in the clinic and verified by measuring urinary sodium and potassium excretion. The potassium content of the diet was increased by 20 mEq/day after 7 days to evaluate the persistence of LCI699 effects in response to further stimulation of aldosterone release. Single doses of LCI699 caused dose-dependent reductions in plasma and urinary aldosterone levels; the maximal inhibition of plasma aldosterone reached a plateau of approximately 80% at the 10 mg single dose, with no further reduction at higher doses up to 200 mg. These results were consistent with those from the Ang-II- and ACTH-stimulation models in rats and monkeys, in which the upper dose of LCI699 also did not completely inhibit aldosterone production (maximal inhibition of 80–90%). Incomplete pharmacologic inhibition of aldosterone synthase by LCI699 at the doses used is the most likely explanation for this observation, because in monkeys a 3000 μg/kg dose of LCI699 decreased plasma aldosterone concentrations to below the detection limit for 24 h (data not shown). Indeed, the steroid profile of patients with genetic aldosterone synthase deficiency is characterized by low to undetectable plasma aldosterone levels and elevated levels of mineralocorticoid precursors (corticosterone or 11-DOC) [16].

LCI699 treatment induced a natriuresis on Day 1 that was similar with all doses and equal to that achieved by eplerenone 100 mg (the approved maximum once-daily dose for use in patients with essential hypertension). The rise in PRA observed after repeated administration of LCI699 reflects the magnitude of RAS counter-regulation following aldosterone synthase inhibition and consequent aldosterone suppression and sodium depletion. LCI699 0.5 mg daily resulted in similar increases in PRA to eplerenone 100 mg, while LCI699 3 mg stimulated markedly larger increases in PRA. The larger dose-dependent counter-regulatory activation of the RAS by LCI699 relative to eplerenone presumably reflects greater suppression of the aldosterone pathway by LCI699 at the higher doses. The consequent increase in levels of Ang II may limit effects of aldosterone synthase inhibition, but this was not tested in the present study.

Repeated once-daily dosing with LCI699 was associated with a resetting of plasma aldosterone levels during the second half of the dosing interval (12–24 h post-dose) followed by a more gradual, chronic increase in baseline aldosterone levels. This pattern probably reflects a combination of the short half-life of LCI699 (approximately 4 h in humans), counter-regulatory stimulation of the RAS, a gradual increase in adrenal aldosterone synthase enzyme, and slow clearance of precursors such as 11-DOC. In the first 12 h after dosing, LCI699 effectively inhibited the activity of aldosterone synthase and plasma aldosterone was decreased. However, during the next 12 h, when LCI699 plasma concentrations had decreased, aldosterone synthase was presumably no longer inhibited. Moreover, the adrenal gland was exposed to elevated levels of renin and Ang II, which stimulate aldosterone release [43]. Thus, with once-daily administration of LCI699 in the morning, the pharmacologic effect of the drug to inhibit aldosterone synthase will essentially have dissipated during the nocturnal rise in ACTH, cortisol and aldosterone. This was the pharmacokinetic and dynamic basis for twice-daily dosing of LCI699 for subsequent studies in patients.

The selectivity of LCI699 for aldosterone synthase over 11β-hydroxylase was evaluated by an ACTH stimulation test in experimental models and in humans. The ACTH test provided safety monitoring for clinically relevant inhibition of glucocorticoid synthesis, which occurred with repeated administration of LCI699 at the 10 mg dose [44]. Measurements of plasma levels of precursors of aldosterone synthesis (11-DOC) and cortisol synthesis (11-deoxycortisol) also provided useful markers of the relative selectivity of aldosterone synthase inhibition. Both precursors are synthesized in the adrenal gland and their production is dependent on normal ACTH levels [45,46]. LCI699 0.5 mg was shown to be selective for inhibition of aldosterone synthase over 11β-hydroxylase throughout the dosing period, as evidenced by reductions in plasma and urinary aldosterone and increases in the aldosterone precursor 11-DOC without effect on basal or ACTH-stimulated cortisol or on the cortisol precursor 11-deoxycortisol. LCI699 1 mg was selective for aldosterone synthase after a single dose on Day 1 and on Day 6 of multiple dose administration, but showed a time-dependent loss of selectivity with continued dosing. Although there was no effect on basal cortisol or 11-deoxycortisol, LCI699 1 mg did result in attenuation of the ACTH-stimulated cortisol response on Day 13. This was confirmed in approximately 20% of hypertensive patients treated with LCI699 1 mg once daily [47]. This lag in pharmacodynamic effect in the absence of a change in plasma pharmacokinetics, may reflect a longer half-life of LCI699 within the adrenals. The 3 mg dose of LCI699 inhibited 11β-hydroxylase on Day 6 (as demonstrated by both an attenuated cortisol response to ACTH and increase in 11-deoxycortisol) and also resulted in a small increase in plasma potassium which, in conjunction with the clinical observations, suggested development of mild hypoaldosteronism.

Several limitations of the human study should be acknowledged. First, although dietary sodium and potassium intake were controlled, there was no assessment of total metabolic sodium and potassium balance during repeated dose administration of LCI699. Second, urinary collections were not fractionated. This likely gave rise to an artifactual apparent attenuation in the effect of LCI699 to inhibit aldosterone synthase when 24-h urinary aldosterone was compared across Days 1, 7 and 14 of administration because of the short half-life of LCI699 and consequent rebound increase in aldosterone 12–24 h after dosing.

There are two potential therapeutic uses of LCI699, as noted by Azizi and colleagues in their review of the therapeutic potential of aldosterone synthase inhibition [33]. First, LCI699 at low doses (below 1 mg) can be used as a selective inhibitor of aldosterone production to treat subsets of patients with hypertension and cardiorenal disease associated with elevated aldosterone levels. In a small, short-term study in 14 patients with primary aldosteronism, LCI699 (0.5 mg and 1 mg) administered twice daily effectively reduced plasma and urinary aldosterone levels and corrected hypokalemia after 4 weeks of treatment. In these low-renin hypertensive patients, reductions in blood pressure were very modest (mean 4 mmHg decrease in ambulatory systolic blood pressure) [48]. In another study of 524 patients with essential hypertension, LCI699 administered at doses between 0.25 and 1 mg resulted in placebo-corrected reductions in ambulatory systolic and diastolic blood pressures of 6–9 mmHg and 3–6 mmHg, respectively after 8 weeks of treatment [39]; ACTH-stimulated cortisol release was suppressed in approximately 20% of subjects receiving LCI699 at a total daily dose of 1 mg in that study. Second, the potency of LCI699 in inhibiting 11β-hydroxylase at daily doses above 3 mg led to further development of LCI699 as a drug candidate for indications in which metyrapone is currently used, both for diagnosis and treatment. Indeed, LCI699 has low pharmacokinetic variability, and may avoid the limitations of metyrapone and other steroidogenesis inhibitors such as ketoconazole and mitotane [49]. In a study in 12 patients with Cushing’s disease, LCI699 2–50 mg twice daily was well tolerated and highly effective in reducing urinary free cortisol (below upper limit of normal in 11 patients) and in lowering blood pressure after 70 days of treatment [19].

## Conclusions

The results of this study of aldosterone synthase inhibition in several cell systems and animal models and their translation to humans provides a rationale and methodology for further investigations to assess the therapeutic potential of new ASIs in chronic diseases characterized by aldosterone excess. Our observations in the dTG rat model suggest that ASIs may be clinically useful in preventing or minimizing the deleterious effects of aldosterone on the heart and kidney and in reducing mortality.

## Additional files

Additional file 1:
**Supplementary methods.**
Additional file 2:
**Pharmacokinetic profile of LCI699 in rat and monkey** ***in vivo*** **models.** Plasma concentration–time profiles for LCI699 after administration in (A) the angiotensin-II-stimulated rat model, (B) the adrenocorticotropic hormone (ACTH)-infusion rat model and (C) the ACTH-stimulated monkey model. Plasma concentrations of LCI699 after administration of the 5 μg/kg dose in monkeys were below the limit of detection. Values are means. Number of evaluable animals was n = 3 per dose group. Additional file 3:
**Effect of LCI699 on Ang-II- and ACTH-stimulated levels of aldosterone and corticosterone in rats.** (A) Mean plasma aldosterone concentrations in the Ang-II-infusion model, and (B) mean plasma aldosterone concentrations and (C) plasma corticosterone concentrations in the ACTH-infusion model after oral administration of LCI699. Ang II infusion (initial loading dose of 300 ng/kg then 100 ng/kg/min) was started 1 h before dosing with LCI699 (time 0 h) and continued until 8 h after dosing. ACTH infusion (initial loading dose of 100 ng/kg then 30 ng/kg/min) was started 1 h before dosing with LCI699 (vertical dotted line; time 0 h) and continued until 8 h after dosing. Values are means. Number of evaluable animals was n = 3 per dose group. Additional file 4:
**Correlation of plasma aldosterone concentrations with cardiac weight, size and function and with renal function.** Correlation of plasma aldosterone concentrations with (A) left ventricular (LV) fractional shortening (FS), (B) LV isovolumic relaxation time (IVRT) as a percentage of RR interval, (C) LV weight normalized to tibial length (LVW/TL), (D) LV myocardial cell size, (E) blood urea nitrogen (BUN) and (F) 24 h urinary albumin excretion (UAE). Each point represents an individual animal, and data are pooled across all dTG rats (controls and LCI699-treated). Measurements were taken at 7 weeks of age. R^2^ and corresponding *P* values are indicated in each panel. Additional file 5:
**Pharmacokinetic profile of LCI699 following a single oral dose in humans.** (A) Plasma concentration–time profile for LCI699 (mg dose of phosphate salt) on Day 1 following single-dose administration in healthy human volunteers. Values are mean ± SD. Number of evaluable subjects is presented in parentheses. Data presented come from the single-dose phase and first dose of the multiple-ascending dose phase; samples were taken only up to 48 h during this phase. (B) Dose-proportionality plot for LCI699. Values (on a logarithmic scale) are mean ± SD area under the plasma concentration–time curve from 0–24 h (AUC_0–24h_) on Day 1. Additional file 6:
**Effect of LCI699 on plasma and urinary cortisol concentrations (human study, multiple-dose phase).** (A) mean plasma cortisol concentrations and (B) percentage change in 24 hour urinary aldosterone from time-matched baseline on Day 1 following administration of LCI699 (0.5, 1 or 3 mg), eplerenone (100 mg) or placebo in healthy human volunteers. Values are mean ± SEM. Number of evaluable subjects for LCI699 0.5, 1, 3, 10 mg, eplerenone and placebo were (plasma) n = 12, 12, 12, 5, 10 and 11 respectively, and (urine) n = 12, 12, 12, 5, 10 and 11 respectively.

## Abbreviations

AAR: Adrenodoxin-adrenodoxin reductase
ACE: Angiotensin-converting enzyme
ACTH: Adrenocorticotropic hormone
ANCOVA: Analysis of covariance
Ang II: Angiotensin II
ANOVA: Analysis of variance
ASIs: Aldosterone synthase inhibitors
AUC: Area under curve
bpm: Beats per minute
BUN: Blood urea nitrogen
C_Cr_: Creatinine clearance
C_max_: Maximum plasma concentrations
CI: Confidence interval
CYP: Cytochrome P450
DBP: Diastolic blood pressure
11-DOC: 11-deoxycorticosterone
dTG: Double-transgenic
EC_50_: Half maximal effective concentration
ED_50_: Half-maximal effective dose
EDTA: Ethylenediaminetetraacetic acid
EIA: Enzyme Immunoassay
ENaC: Epithelial sodium channel
fps: Frames per second
FS: Fractional shortening
HR: Heart rate
IC_50_: Half maximal inhibitory concentration
i.v.: Intravenous
IVRT: Isovolumic relaxation time
K_i_: Inhibitor constant
LC-MS/MS: Liquid chromatography-tandem mass spectrometry
LV: Left ventricular
LVW/TL: LV weight normalized to tibial length
MAP: Mean arterial pressure
MRAs: Mineralocorticoid receptor antagonists
PAC: Plasma aldosterone concentration
PRA: Plasma renin activity
RALES: Randomized ALdactone Evaluation Study
RAS: Renin-angiotensin system
SBP: Systolic blood pressure
SD: Standard deviation
S-D: Sprague-Dawley
SEM: Standard error of the mean
*t*_½_: Half-life
TDI: Time delay and integration
*t*_max_: Time to maximum plasma concentration
UAE: Urinary albumin excretion
UAldoE: Urinary aldosterone excretion
VAP: Vascular access port

## References

[1] Williams GH (1994). Essential hypertension as an endocrine disease. Endocrinol Metab Clin North Am.

[2] Joffe HV, Adler GK (2005). Effect of aldosterone and mineralocorticoid receptor blockade on vascular inflammation. Heart Fail Rev.

[3] Pitt B, Zannad F, Remme WJ, Cody R, Castaigne A, Perez A, Palensky J, Wittes J (1999). The effect of spironolactone on morbidity and mortality in patients with severe heart failure. Randomized Aldactone Evaluation Study Investigators. N Engl J Med.

[4] Pitt B, Remme W, Zannad F, Neaton J, Martinez F, Roniker B, Bittman R, Hurley S, Kleiman J, Gatlin M (2003). Eplerenone, a selective aldosterone blocker, in patients with left ventricular dysfunction after myocardial infarction. N Engl J Med.

[5] Zannad F, McMurray JJ, Krum H, van Veldhuisen DJ, Swedberg K, Shi H, Vincent J, Pocock SJ, Pitt B (2011). Eplerenone in patients with systolic heart failure and mild symptoms. N Engl J Med.

[6] Laragh JH (1973). Vasoconstriction-volume analysis for understanding and treating hypertension: the use of renin and aldosterone profiles. Am J Med.

[7] Ramsay LE, Hettiarachchi J, Fraser R, Morton JJ (1980). Amiloride, spironolactone, and potassium chloride in thiazide-treated hypertensive patients. Clin Pharmacol Ther.

[8] Chun TY, Pratt JH (2006). Nongenomic renal effects of aldosterone: dependency on NO and genomic actions. Hypertension.

[9] Mihailidou AS, Funder JW (2005). Nongenomic effects of mineralocorticoid receptor activation in the cardiovascular system. Steroids.

[10] Wehling M (1997). Specific, nongenomic actions of steroid hormones. Annu Rev Physiol.

[11] Struthers AD (1995). Aldosterone escape during ACE inhibitor therapy in chronic heart failure. Eur Heart J.

[12] Staessen J, Lijnen P, Fagard R, Verschueren LJ, Amery A (1981). Rise in plasma concentration of aldosterone during long-term angiotensin II suppression. J Endocrinol.

[13] Taves MD, Gomez-Sanchez CE, Soma KK (2011). Extra-adrenal glucocorticoids and mineralocorticoids: evidence for local synthesis, regulation, and function. Am J Physiol Endocrinol Metab.

[14] Silvestre JS, Heymes C, Oubenaissa A, Robert V, Aupetit-Faisant B, Carayon A, Swynghedauw B, Delcayre C (1999). Activation of cardiac aldosterone production in rat myocardial infarction: effect of angiotensin II receptor blockade and role in cardiac fibrosis. Circulation.

[15] White PC (2004). Aldosterone synthase deficiency and related disorders. Mol Cell Endocrinol.

[16] Veldhuis JD, Melby JC (1981). Isolated aldosterone deficiency in man: acquired and inborn errors in the biosynthesis or action of aldosterone. Endocr Rev.

[17] Meredith EL, Ksander G, Monovich LG, Papillon JP, Liu Q, Miranda K, Morris P, Rao C, Burgis R, Capparelli M, Hu QY, Singh A, Rigel DF, Jeng AY, Beil M, Fu F, Hu CW, LaSala D (2013). Discovery and in vivo evaluation of potent dual CYP11B2 (Aldosterone Synthase) and CYP11B1 inhibitors. ACS Med Chem Lett.

[18] Hakki T, Bernhardt R (2006). CYP17- and CYP11B-dependent steroid hydroxylases as drug development targets. Pharmacol Ther.

[19] Bertagna X, Pivonello R, Fleseriu M, Zhang Y, Robinson P, Taylor A, Watson CE, Maldonado M, Hamrahian AH, Boscaro M, Biller BM (2014). LCI699, a potent 11beta-hydroxylase inhibitor, normalizes urinary cortisol in patients with Cushing’s disease: results from a multicenter, proof-of-concept study. J Clin Endocrinol Metab.

[20] LaSala D, Shibanaka Y, Jeng AY (2009). Coexpression of CYP11B2 or CYP11B1 with adrenodoxin and adrenodoxin reductase for assessing the potency and selectivity of aldosterone synthase inhibitors. Anal Biochem.

[21] Rigel DF, Fu F, Beil M, Hu CW, Liang G, Jeng AY (2010). Pharmacodynamic and pharmacokinetic characterization of the aldosterone synthase inhibitor FAD286 in two rodent models of hyperaldosteronism: comparison with the 11beta-hydroxylase inhibitor metyrapone. J Pharmacol Exp Ther.

[22] Bohlender J, Fukamizu A, Lippoldt A, Nomura T, Dietz R, Menard J, Murakami K, Luft FC, Ganten D (1997). High human renin hypertension in transgenic rats. Hypertension.

[23] St-Jacques R, Toulmond S, Auger A, Binkert C, Cromlish W, Fischli W, Harris J, Hess P, Jie L, Liu S, Riendeau D, Steiner B, Percival MD (2011). Characterization of a stable, hypertensive rat model suitable for the consecutive evaluation of human renin inhibitors. J Renin Angiotensin Aldosterone Syst.

[24] Gough K, Hutchison M, Keene O, Byrom W, Ellis S, Lacey L, McKellar J (1995). Assessment of dose proportionality: report from the Statisticians in the Pharmaceutical Industry/Pharmacokinetics UK Joint Working Party. Drug Inf J.

[25] Cai TQ, Stribling S, Tong X, Xu L, Wisniewski T, Fontenot JA, Struthers M, Akinsanya KO: **Rhesus monkey model for concurrent analyses of in vivo selectivity, pharmacokinetics and pharmacodynamics of aldosterone synthase inhibitors.** *J Pharmacol Toxicol Methods* 2014, doi:10.1016/j.vascn.2014.09.011. 10.1016/j.vascn.2014.09.011 25304940

[26] Hu Q, Yin L, Hartmann RW: **Aldosterone synthase inhibitors as promising treatments for mineralocorticoid dependent cardiovascular and renal diseases.** *J Med Chem* 2014, e-pub ahead of print, doi:10.1021/jm401430e. 10.1021/jm401430e 24422519

[27] Berl T, Linas SL, Aisenbrey GA, Anderson RJ (1977). On the mechanism of polyuria in potassium depletion. The role of polydipsia. J Clin Invest.

[28] Fiebeler A, Nussberger J, Shagdarsuren E, Rong S, Hilfenhaus G, Al-Saadi N, Dechend R, Wellner M, Meiners S, Maser-Gluth C, Jeng AY, Webb RL, Luft FC, Muller DN (2005). Aldosterone synthase inhibitor ameliorates angiotensin II-induced organ damage. Circulation.

[29] Min LJ, Mogi M, Li JM, Iwanami J, Iwai M, Horiuchi M (2005). Aldosterone and angiotensin II synergistically induce mitogenic response in vascular smooth muscle cells. Circ Res.

[30] Montezano AC, Callera GE, Yogi A, He Y, Tostes RC, He G, Schiffrin EL, Touyz RM (2008). Aldosterone and angiotensin II synergistically stimulate migration in vascular smooth muscle cells through c-Src-regulated redox-sensitive RhoA pathways. Arterioscler Thromb Vasc Biol.

[31] Gomez-Sanchez EP (2010). The mammalian mineralocorticoid receptor: tying down a promiscuous receptor. Exp Physiol.

[32] Yin L, Hu Q, Emmerich J, Lo MM, Metzger E, Ali A, Hartmann RW (2014). Novel pyridyl- or isoquinolinyl-substituted indolines and indoles as potent and selective aldosterone synthase inhibitors. J Med Chem.

[33] Azizi M, Amar L, Menard J (2013). Aldosterone synthase inhibition in humans. Nephrol Dial Transplant.

[34] Berger S, Bleich M, Schmid W, Cole TJ, Peters J, Watanabe H, Kriz W, Warth R, Greger R, Schutz G (1998). Mineralocorticoid receptor knockout mice: pathophysiology of Na+ metabolism. Proc Natl Acad Sci U S A.

[35] Bleich M, Warth R, Schmidt-Hieber M, Schulz-Baldes A, Hasselblatt P, Fisch D, Berger S, Kunzelmann K, Kriz W, Schutz G, Greger R (1999). Rescue of the mineralocorticoid receptor knock-out mouse. Pflugers Arch.

[36] Makhanova N, Sequeira-Lopez ML, Gomez RA, Kim HS, Smithies O (2006). Disturbed homeostasis in sodium-restricted mice heterozygous and homozygous for aldosterone synthase gene disruption. Hypertension.

[37] O’Neil RG, Helman SI (1977). Transport characteristics of renal collecting tubules: influences of DOCA and diet. Am J Physiol.

[38] Peterson LN, Wright FS (1977). Effect of sodium intake on renal potassium excretion. Am J Physiol.

[39] Calhoun DA, White WB, Krum H, Guo W, Bermann G, Trapani A, Lefkowitz MP, Menard J (2011). Effects of a novel aldosterone synthase inhibitor for treatment of primary hypertension: results of a randomized, double-blind, placebo- and active-controlled phase 2 trial. Circulation.

[40] Brown R, Quirk J, Kirkpatrick P (2003). Eplerenone. Nat Rev Drug Discov.

[41] Mihailidou AS, Le Loan TY, Mardini M, Funder JW (2009). Glucocorticoids activate cardiac mineralocorticoid receptors during experimental myocardial infarction. Hypertension.

[42] Fischer R, Dechend R, Gapelyuk A, Shagdarsuren E, Gruner K, Gruner A, Gratze P, Qadri F, Wellner M, Fiebeler A, Dietz R, Luft FC, Muller DN, Schirdewan A (2007). Angiotensin II-induced sudden arrhythmic death and electrical remodeling. Am J Physiol Heart Circ Physiol.

[43] Young DB (1988). Quantitative analysis of aldosterone’s role in potassium regulation. Am J Physiol.

[44] Kazlauskaite R, Evans AT, Villabona CV, Abdu TA, Ambrosi B, Atkinson AB, Choi CH, Clayton RN, Courtney CH, Gonc EN, Maghnie M, Rose SR, Soule SG, Tordjman K (2008). Corticotropin tests for hypothalamic-pituitary- adrenal insufficiency: a metaanalysis. J Clin Endocrinol Metab.

[45] Coppage WS, Island D, Smith M, Liddle GW (1959). Inhibition of aldosterone secretion and modification of electrolyte excretion in man by a chemical inhibitor of 11 beta-hydroxylation. J Clin Invest.

[46] Liddle GW, Island D, Lance EM, Harris AP (1958). Alterations of adrenal steroid patterns in man resulting from treatment with a chemical inhibitor of 11 beta-hydroxylation. J Clin Endocrinol Metab.

[47] White WB, Calhoun DA, Krum H, Guo W, Trapani AJ, Lefkowitz M, Menard J (2010). Blockade of aldosterone production as a novel approach to the management of high blood pressure: efficacy and tolerability of the aldosterone synthase inhibitor LCI699 in patients with stage 1-2 hypertension. J Am Coll Cardiol.

[48] Amar L, Azizi M, Menard J, Peyrard S, Watson C, Plouin PF (2010). Aldosterone synthase inhibition with LCI699: a proof-of-concept study in patients with primary aldosteronism. Hypertension.

[49] Biller BM, Grossman AB, Stewart PM, Melmed S, Bertagna X, Bertherat J, Buchfelder M, Colao A, Hermus AR, Hofland LJ, Klibanski A, Lacroix A, Lindsay JR, Newell-Price J, Nieman LK, Petersenn S, Sonino N, Stalla GK, Swearingen B, Vance ML, Wass JA, Boscaro M (2008). Treatment of adrenocorticotropin-dependent Cushing’s syndrome: a consensus statement. J Clin Endocrinol Metab.

