# Supplementary material for: Aldosterone synthase inhibition: cardiorenal protection in animal disease models and translation of hormonal effects to human subjects
Source: J Transl Med. 2014 Dec 10;12:340. doi: 10.1186/s12967-014-0340-9 (PMC4301837; doi:10.1186/s12967-014-0340-9)
Supplement: Additional file 1: — Supplementary methods. [file 12967_2014_340_MOESM1_ESM.docx]

# Supplementary methods

### **Surgical preparation**

Monkeys were anesthetized with medetomidine (0.1 mg/kg i.m.) and ketamine (3 mg/kg i.m.), intubated with an endotracheal tube, and maintained on isoflurane (2% in 1–2 l/min oxygen). After the animal was sedated, meloxicam (0.4 mg/kg s.c.) and buprenorphine (0.01 mg/kg i.m.) were administered for pre-emptive analgesia. Cefazolin (20 mg/kg i.m.) was administered at the time of surgery and twice daily for the first 3 post-operative days. Catheters (3.5 or 5 French polyurethane Hydrocoat; Access Technologies, Skokie, IL, USA) were surgically and chronically inserted into a femoral vein and attached to vascular access ports (VAP; Access Technologies) that were subcutaneously implanted in the mid-dorsal region. Post-operatively, animals were given atipamezole (0.225 mg/kg i.m.) to reverse the medetomidine sedation, and at least one more injection of meloxicam (0.02 mg/kg s.c. or orally) and of buprenorphine (0.01 mg/kg i.m.) was administered for additional post-operative analgesia. VAPs were flushed and locked with 200 U/ml heparin in saline, flushed daily for the first post-operative week, and then flushed and locked weekly with 200 U/ml heparin plus urokinase 2000 U/ml (Abbokinase, ImaRx Therapeutics, Inc., Tucson, AZ, USA) thereafter.

### **Measurement of aldosterone, cortisol and corticosterone concentrations**

### In vitro analyses

Aldosterone concentration was measured using 96-well plates. Each test sample was incubated with 0.02 µCi D-[1,2,6,7-^3^H(N)]aldosterone (PerkinElmer, Boston, MA, USA) and 0.2 µg anti-aldosterone antibody (ATCC, Manassas, VA, USA) in phosphate-buffered saline containing 0.1% Triton X-100, 0.1% bovine serum albumin, and 12% glycerol (in a total volume of 200 µL) at room temperature for 1 h. Anti-mouse polyvinyltoluene (PVT) scintillation proximity assay (SPA) beads (50 µL: GE Health Sciences, Piscataway, NJ, USA) were then added to each well and incubated overnight at room temperature prior to counting in a Microbeta plate counter (PerkinElmer). Determination of cortisol and corticosterone concentrations followed a similar protocol. Human and monkey samples were incubated with 0.02 µCi [1,2,6,7-^3^H(N)]hydrocortisone (PerkinElmer) and 0.3 µg anti-cortisol antibody (US Biological, Swampscott, MA, USA), while the rat assay used 0.02 µCi [1, 2, 6, 7-^3^H(N)]corticosterone and 0.2 µg anti-corticosterone antibody (US Biological) and anti-sheep PVT SPA beads (GE Health Sciences). The amount of aldosterone, cortisol or corticosterone was calculated by comparison with a standard curve.

*Analyses of blood samples from in vivo studies*

Blood samples were centrifuged at 20,000*g* for 20 min at 4°C to separate out plasma, which was aliquoted and frozen at −70°C until required for analysis. Plasma aldosterone and cortisol concentrations from animal studies were measured using commercially available solid-phase ^125^I radioimmunoassay kits (Coat-A-Count Aldosterone TKAL1; Coat-A-Count Cortisol TKCO1; Siemens Healthcare Diagnostic Products).

*Analyses of blood samples from human pharmacokinetic studies*

Aldosterone levels were measured by radioimmunoasssay with 100 μl of EDTA plasma or 500 μl of urine using the DSL-8600 ACTIVE^®^ Aldosterone Coated-Tube radioimmunoassay kit from Diagnostic Systems Laboratories, Inc. (LLOQ was 69 pmol/l [25.0 pg/ml] for plasma and 2 nmol/l [0.75 ng/ml] for urine).

Cortisol, 11-deoxycortisol and 11-deoxycorticosterone levels were measured by LC/MS/MS of 250 μL of EDTA plasma or 500 μL urine (LLOQ was 13.79 nmol/L [5.00 ng/mL] for cortisol and 2.89 nmol/L [1.00 ng/mL] for 11-deoxycortisol and 11-deoxycorticosterone in plasma and 2.76 nmol/L [1.00 ng/mL] for cortisol in urine). For plasma cortisol, based on 9 standards ranging from 5–300ng/ml (>70 replicates of each) run at the time of sample analysis, the coefficient of variation (CV) and were 2.14–5.32% and 97.6–104%, respectively. For urine cortisol, based on 7 standards ranging from 1–100ng/ml (22 replicates of each) run at the time of sample analysis, CV and accuracy were 1.55–4.22% and 97.0–101.6%, respectively. For plasma 11-deoxycortisol, based on 9 standards ranging from 1–300ng/ml (>50 replicates of each) run at the time of sample analysis, CV and accuracy were 3.69–8.22% and 91.33–108%, respectively. For 11-deoxycorticosterone, based on 8 standards ranging from 0.05–1 ng/ml (19–20 replicates of each) run during assay validation, CV of the plasma assay was 2.38–8.71% and bias was -2.4–4.0%.

ACTH was measured in 200 μl of EDTA plasma using the ELISA-ACTH kit from Cis Bio International (LLOQ was 0.44 pmol/l [2 pg/ml]). PRA was measured by radioimmunoassay of Ang I generation in 500 μl of EDTA plasma using the REN-CT2 kit from Cis Bio International (LLOQ was 0.2 ng/ml).

**Measurement of LCI699 concentration**

The concentration of LCI699 was measured by a liquid chromatography separation coupled with tandem mass spectrometric detection (LC/MS/MS). The LLOQ for this assay was 0.22 nmol/l (0.05 ng/ml) in plasma and 4.4 nmol/l (1 ng/ml) in urine. Concentrations of LCI699 below the LLOQ were treated as zero for the calculation of concentration means and pharmacokinetic parameters.
